# Self‐Organized Vascularized Human Liver Spheroids: Serum‐Free Culture Conditions and Use as Tissue Building Blocks

**DOI:** 10.1002/smll.74536

**Published:** 2026-07-19

**Authors:** Alina Filatova, Olivera Valentirović Dann, Jennifer B. Schwarzkopf, Tengku I. Maulana, Jamina S. Gerhardus, Leon Kaysan, Katja Meier, Andreas Blaeser, Beate K. Straub, Holger Gerhardt, Ulrike A. Nuber

**Affiliations:** ^1^ Stem Cell and Development Biology, Department of Biology Technical University of Darmstadt Germany; ^2^ Max Delbrück Center for Molecular Medicine in the Helmholtz Association Berlin Germany; ^3^ DZHK (German Center for Cardiovascular Research) Berlin Germany; ^4^ Charité ‐ Universitätsmedizin Berlin Berlin Germany; ^5^ Institute for BioMedical Printing Technology, Department of Mechanical Engineering Technical University of Darmstadt Germany; ^6^ Centre for Synthetic Biology Technical University of Darmstadt Germany; ^7^ Tissue Biobank of the University Medical Center Mainz Germany; ^8^ Institute of Pathology University Medical Center of the Johannes Gutenberg‐University Mainz Germany; ^9^ Berlin Institute of Health Berlin Germany; ^10^ Department of Mechanical Engineering Technical University of Darmstadt Germany

**Keywords:** endothelial cells, HepaRG, hepatocyte, HUVEC, serum replacement, spheroid, tissue engineering

## Abstract

Engineering vascularized human liver tissue for in vitro and in vivo applications remains a major challenge. Here, we describe a scalable approach to generate human liver spheroids with self‐organized, lumen‐containing vascular networks and demonstrate their use as building blocks for fabricating vascularized single and multilayer tissue constructs. Spheroids were formed from HepaRG liver cells, human umbilical vein endothelial cells, and adipose tissue‐derived mesenchymal stem cells. Including the latter in specific ratios prevented a spatial segregation of hepatic and endothelial compartments, enabling endothelial network formation. We present two media for culturing these spheroids: a serum‐reduced medium and a defined serum‐free medium containing Gibco KnockOut Serum Replacement. These media supported the long‐term maintenance of hepatocytes in a metabolically active, relatively mature state, as well as the persistence of endothelial networks. Spheroid‐derived endothelial cells established anastomoses with external endothelial channels in microfluidic devices, and upon grafting into mouse liver tissue extended into the host parenchyma. Moreover, endothelial sprouts emerging from the spheroids formed inter‐spheroid connections within permissive hydrogels, a process that depended on the inter‐spheroid distance. Finally, we demonstrate the fabrication of planar tissue layers with vascularly interconnected spheroids and the creation of a macroscale tissue construct from several such layers.

## Graphical abstract

This approach addresses a major challenge in tissue engineering: creating larger, vascularized tissues. Microvascular networks in liver spheroids are developed through self‐organization mediated by mesenchymal stem cells. These spheroids are maintained in serum‐reduced or serum‐free medium and serve as building blocks to form planar tissue layers featuring inter‐spheroid vascular connectivity. Employing a biocompatible membrane, such layers are stacked into multi‐layered tissue.

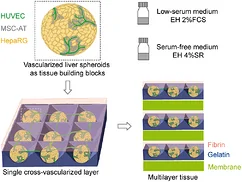

**Abbreviations**
3D: three‐dimensional
bFGF: basic fibroblast growth factor
BM: basal medium
CollMet: collagen–methylcellulose
CYP3A4: cytochrome P450 family 3 subfamily A member 4
dPBS: Dulbecco's Phosphate‐Buffered Saline
EH 2%FCS medium: Endothelial cell Hepatocyte medium with 2% FCS
EH 4%SR medium: Endothelial cell Hepatocyte medium with 4% KnockOut Serum Replacement
FibAgGel: fibrin‐agarose–gelatin
FCS: fetal calf serum
HNF4α: hepatocyte nuclear factor 4α
h: hour
hPSCs: human pluripotent stem cells
IGF‐1 LR3: insulin‐like growth factor 1 long R3
HUVECs: human umbilical vein endothelial cells
min: minute
MSCs: mesenchymal stem cells
PAS staining: periodic acid‐Schiff staining
PFA: paraformaldehyde
RT: room temperature
SR: KnockOut Serum Replacement

## 1 Introduction

Human tissues generated in the laboratory provide versatile in vitro systems for studying human diseases, drug development, and toxicology assessment, and hold great promise for regenerative medicine [1, 2]. They have the potential to reduce animal testing and to serve as an alternative to donated human tissue. Among human tissue types, liver tissue stands out as particularly relevant for drug development, toxicology assessment, and the replacement or compensation of lost or impaired liver function through tissue transplantation or extracorporeal application. The high medical demand for human liver tissue is also due to critical differences in metabolism and disease characteristics between human and animal liver cells. Drug‐induced liver injury is the most frequent cause of the withdrawal of an approved drug, accounting for 30% of all cases, and the insufficient ability of animal models to reflect human liver pathophysiology and drug responses is an important factor for this high failure rate [3]. There is a high medical need for human liver tissue to compensate for or replace dysfunctional one in several severe diseases: chronic liver failure due to chronic viral hepatitis, long‐term alcohol abuse, non‐alcoholic fatty liver disease, autoimmune hepatitis, and genetic or metabolic disorders. Acute liver failure, often caused by viral hepatitis or toxic agents, as well as inherited liver‐based metabolic dysfunction, further contribute to this demand [4, 5, 6]. However, a routine application of laboratory‐generated human liver tissue is hindered by several limitations. One critical deficit of current human tissue models, including those of the liver, is their lack of a vascular system, which severely restricts their utility for various reasons: (i) interactions between vascular and tissue‐specific cells are required for normal tissue homeostasis and function and are impaired in various liver diseases, (ii) the vascular system mediates the entry of drugs and pathogens into liver tissue, (iii) a perfusable vascular system is required for tissue models with diameters exceeding the oxygen diffusion limit, i.e., of more than only a few hundred µm. Another key limitation of current human tissue models is their scalability. This is particularly relevant for therapeutic applications of human liver tissue, since sufficiently high tissue mass is needed to compensate for lost function. Bioprinting processes of single cells are currently too slow to fabricate tissue of clinically relevant scale within an acceptable time frame [7]. One appealing strategy to rapidly fabricate larger tissue formations is their assembly from tissue building blocks that already consist of hundreds to thousands of cells [7, 8]. For standardized fabrication, such building blocks should ideally have a controllable and uniform size, allow spatially defined deposition and assembly, and be suitable for large‐scale production. These requirements are met by spherical three‐dimensional (3D) cell cultures (spheroids and organoids) with diameters of only a few hundred micrometers. At this size, oxygen and nutrients can still be supplied by the surrounding medium during their production and maintenance, and such 3D cell cultures can principally fuse into larger assemblies [7, 9].

The term spheroid, as first described in the 1970s, is mainly used in the literature for spherical 3D cell formations that result from cell aggregation and division [10]. Spheroids can consist of single or multiple cell types. Organoids develop from stem cells through self‐organizing processes and typically consist of different stem cell‐derived cell types; the term organoid implies that these 3D cell formations recapitulate specific structural and functional properties of an organ [11]. Many organoid generation protocols produce spherical cell formations, including some that resemble human liver tissue [12]. Of note, the principal applicability of human liver tissue‐like spheroids or organoids to restore or replace lost liver functions has been shown upon transplantation into mice [13, 14, 15], though much larger constructs would be required for human applications. Since the survival of larger tissue constructs after transplantation depends on rapid vascularization within the tissue, strategies to generate pre‐vascularized tissue building blocks are being pursued. Using different approaches, the formation of endothelial cell networks within 3D human liver‐like tissue has been shown. However, in very few cases, vascular‐like structures that contain a lumen formed by endothelial cells have been achieved, which is a prerequisite for their perfusability (for review, see [16]). In most vascularized organoids or spheroids, luminal structures—i.e., endothelial tubes with a hollow interior—were only observed after transplantation into mice. In these cases, only low numbers of 3D cultures were transplanted and not assembled into larger tissue formations (e.g., [17, 18]). Harrison, Siller, Tanaka, Chollet et al. demonstrated endothelial structures with a lumen in liver organoids produced in vitro from human pluripotent stem cells (hPSCs) according to light microscopy [19]. Kim and colleagues reported luminal vasculature in liver organoids generated from hPSCs based on in vitro live perfusion, light and transmission electron microscopy data [20, 21]. Here, we identify various parameters that govern the self‐organization of endothelial cells into lumen‐containing vascular structures with key hallmarks of natural capillaries inside human 3D liver spheroids. We show that human umbilical vein endothelial cells (HUVECs), the most frequently used human endothelial cell type employed in vascularization approaches of 3D cell cultures, do not form a network upon aggregation with human bipotent HepaRG liver cells into spheroids unless an additional mesenchymal cell type is integrated. Furthermore, our experiments demonstrate that defined cell ratios of these three cell types during aggregation into 3D cell clusters allow the formation of vascular structures. In addition, we developed novel serum‐reduced and serum‐free cell culture media that sustain the long‐term maintenance of functional hepatocytes together with HUVEC‐based vascular structures. Moreover, we demonstrate the principal applicability of vascularized liver spheroids as tissue building blocks for the generation of larger tissue constructs, forming endothelial connections between the spheroids and with larger endothelialized channels. Finally, we present a methodology for generating tissue layers from evenly distributed spheroids that interconnect via vascular sprouts, and the fabrication of macroscale tissue constructs from multiple such layers.

## 2 Experimental Section

### 2.1 Cell Culture Experiments

#### 2.1.1 Cells and Media

All cells were maintained at 37°C in a humidified 5% CO_2_ incubator. Cryopreserved HepaRG cells (HPR101, Biopredic International, Saint Gregoire, France) were cultured in HepaRG medium (William's E medium (22551022, Thermo Fisher Scientific, Schwerte, Germany) with 10% fetal calf serum (FCS), 5 µg/mL insulin (I9278, Sigma‐Aldrich, Darmstadt, Germany), 50 µM hydrocortisone 21‐hemisuccinate sodium salt (sc‐250130, Santa Cruz Biotechnology, Heidelberg, Germany), 2 mM L‐glutamine, 100 U/mL penicillin, and 0.1 mg/mL streptomycin). GFP‐labeled human umbilical vein endothelial cells (HUVECs) (ZHC‐2402, Cellworks, South San Francisco, USA) were maintained in Endothelial Cell Growth Medium 2 (ECGM2 2%FCS, C‐22011, PromoCell, Heidelberg, Germany) with 100 U/mL penicillin and 0.1 mg/mL streptomycin. Human mesenchymal stem cells from adipose tissue (MSCs, C‐12977, Promocell) were grown in Mesenchymal Stem Cell Growth Medium 2 (C‐28009, Promocell) with 100 U/mL penicillin and 0.1 mg/mL streptomycin. All cell lines were weekly tested for mycoplasma contamination and were confirmed to be mycoplasma‐negative.

#### 2.1.2 Generation of Master Stamps

The master stamps were generated according to Dahlmann et al. [22] by pouring 2.5 mL of silicone (Hydrosil components A and B mixed 1:1, 101301, Siladent Dr. Böhme & Schoeps GmbH, Goslar, Germany) into an AggreWell800 plate (34811, Stemcell Technologies, Saint‐Égréve, France) used as template. Air bubbles were removed from the liquid silicone by centrifugation at 55 × g for 10 s using an Eppendorf 5810R centrifuge equipped with a swing‐bucket rotor (Eppendorf, Cologne, Germany). The solidified silicone master stamps were gently removed from the AggreWells, washed with cell culture‐grade water and 80% ethanol, sterilized by incubation under UV light for 30 minutes (min), and air‐dried under sterile conditions in a laminar flow hood.

#### 2.1.3 Generation of Spheroids

**FIGURE 1:**
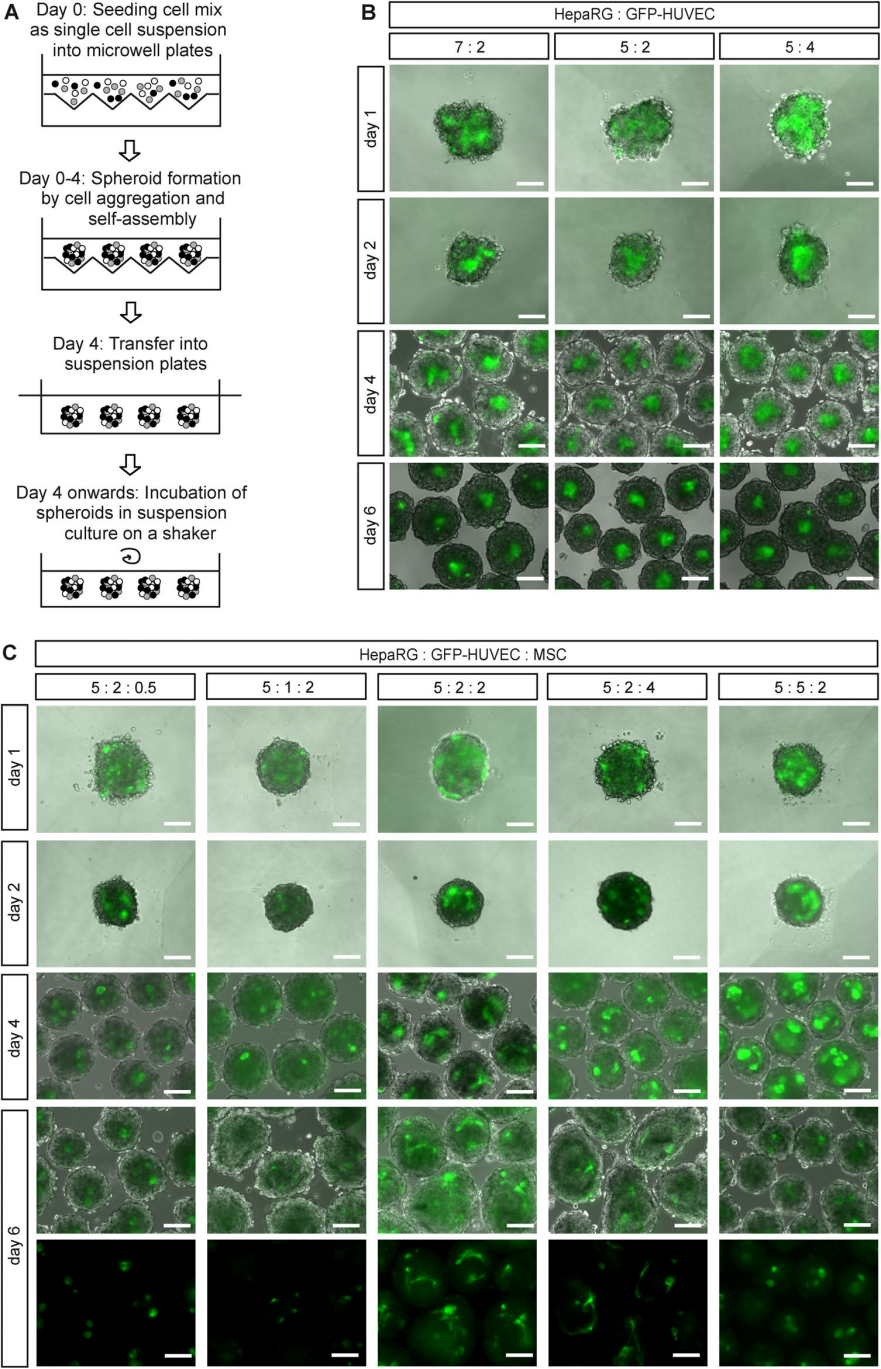
Mesenchymal stem cells facilitate endothelial cell network formation within human liver cell spheroids. (A) Experimental workflow. Mixed single cells in ECGM2 2%FCS medium were seeded into agarose microwell plates, that were generated using a negative silicone stamp. After 4 days, the assembled spheroids were transferred into suspension plates and kept on a shaker. (B) Merged bright‐field and GFP fluorescence microscopy images of spheroids composed of two cell types (HepaRG cells and GFP‐HUVECs) seeded at indicated ratios and analyzed on days 1, 2, 4, and 6. All spheroids were cultured in ECGM 2%FCS medium. Scale bars: 100 µm. (C) Merged bright‐field and fluorescence microscopy images of spheroids composed of three cell types (HepaRG, GFP‐HUVEC, and MSC) seeded at indicated ratios and analyzed on days 1, 2, 4, and 6. For day 6, fluorescence images only are shown in addition (bottom row). All spheroids were cultured in ECGM 2%FCS medium. Scale bars: 100 µm.

Pre‐heated solution of 2% agarose in William's E medium was filled into wells of a 12‐well plate, and the master stamps were pressed into the agarose (see Figure 1A). After incubation for 20 min at 4°C, the stamps were removed, and the plate was sterilized for 30 min under UV light. The cells were seeded into the agarose wells at indicated ratios using 1.2 × 10^6^ cells per well in 2 mL of medium with 50 µg/mL gentamicin and incubated for 4 days in the cell culture incubator to form aggregates. To generate HepaRG monoculture spheroids, 1.2 × 10^6^ HepaRG cells per well were used, and the same protocol was applied. It is expected that approximately 300 spheroids with 4000 cells each are generated per well. Thereafter, the spheroids were transferred in 2 mL of fresh medium into a 6‐well suspension plate coated with Anti‐Adherence Rinsing Solution (07010, Stemcell Technologies). Changes of the entire medium were performed twice a week; 2.5 mL medium were used per well. Phase‐contrast and GFP fluorescence images of the cultured spheroids were taken using a Zeiss Observer D1 microscope.

In some experiments, the spheroids were cultured in EH 2%FCS medium consisting of 47.75% of Endothelial Cell Basal Medium 2 (C‐22211, Promocell), 47.75% of William's E medium (22551022, Thermo Fisher Scientific), 5 ng/mL EGF (recombinant human), 10 ng/mL basic fibroblast growth factor (bFGF, recombinant human), 20 ng/mL insulin‐like growth factor 1 long R3 (IGF‐1 LR3, recombinant human), 0.5 ng/mL VEGF165 (recombinant human), 1 µg/mL ascorbic acid, 22.5 µg/mL heparin, 0.2 µg/mL hydrocortisone, 2% FCS (all from the bullet kit C‐22111, Promocell), 5 µg/mL insulin 21‐hemisuccinate sodium salt (I9278, Sigma‐Aldrich), 2 mM L‐glutamine, 100 U/mL penicillin, and 0.1 mg/mL streptomycin, or EH 4%SR medium containing 46.75% of Endothelial Cell Basal Medium 2 (C‐22211, Promocell), 46.75% of William's E medium (12551, Thermo Fisher Scientific), 5 ng/mL EGF, 10 ng/mL bFGF, 20 ng/mL IGF‐1 LR3, 0.5 ng/mL VEGF165, 1 µg/mL ascorbic acid, 22.5 µg/mL heparin, 0.2 µg/mL hydrocortisone (all from the bullet kit C‐22111, Promocell), 5 µg/mL insulin 21‐hemisuccinate sodium salt (I9278, Sigma‐Aldrich), 4% Gibco KnockOut Serum Replacement (SR, 10828028, Thermo Fisher Scientific, SR), 2 mM L‐glutamine, 100 U/mL penicillin, and 0.1 mg/mL streptomycin.

#### 2.1.4 Cultivation of Adherent Cells for Growth Assessment

0.3 × 10^6^ HepaRG, GFP‐HUVEC, or MSC cells per well of a 6‐well plate were seeded in triplicate in 2 mL of the corresponding cell type‐specific medium (HepaRG medium, Endothelial Cell Growth Medium 2, or Mesenchymal Stem Cell Growth Medium 2, respectively) or in EH 2%FCS. 48 h later, cell morphologies were assessed using a Zeiss Observer D1 microscope.

#### 2.1.5 Embedding and Culture of Vascularized Spheroids in Microfluidic Chips

Triple‐cell spheroids were generated 5 days prior to embedding, according to the protocol above. The spheroids embedded in fibrin gel were seeded into the central chamber of the idenTX3 chip (Aim Biotech, Nucleos, Singapore). RFP‐labeled HUVECs (P20216, Innoprot, Bizkaia, Spain) were loaded into one side channel of the chip to form a confluent monolayer acting as parent vessel for the angiogenesis assay. Human brain pericytes (P10363, Innoprot) were seeded into the opposing side channel. The cells were cultured in EH 2%FCS or EH 4%SR medium for 4–5 days, with 50 ng/mL VEGF165 added to the pericyte‐containing side channel to establish a VEGF165 gradient and thereby induce sprouting angiogenesis.

#### 2.1.6 Preparation of Spheroids for Embedding and Culture of Spheroids in Hydrogels

The generation of spheroids was initiated 5 days prior to embedding according to the protocol above. For embedding, spheroids were washed with calcium‐ and magnesium‐free Dulbecco's Phosphate‐Buffered Saline (dPBS) (D8537‐500ML, Sigma‐Aldrich) and resuspended in basal medium (BM), composed of a 1:1 mixture of William's E Medium and Endothelial Cell Basal Medium 2 (C‐22211, PromoCell, Heidelberg, Germany). 15–30 spheroids were transferred into each well of a flat‐bottom 96‐well plate (96‐wp) and kept at 37°C until gel casting. The spheroids were embedded into hydrogels as specified below and cultured in 200 µL of EH 2%FCS or EH 4%SR medium supplemented with 25 ng/mL human VEGF165 (100‐20‐2UG, PeproTech Thermo Fisher Scientific, Schwerte, Germany). The medium was changed every 1–2 days. Three independent experiments were performed, each with three replicates.

#### 2.1.7 Fibrin Gel Preparation and Spheroid Embedding

Fibrin gels contained 5 mg/mL fibrinogen (F4883, Sigma‐Aldrich), 6 U/mL thrombin (605157‐1KU, Sigma‐Aldrich), and 42.5% BM. A 15 mg/mL stock solution of fibrinogen (F4883, Sigma‐Aldrich) was prepared in 0.9% NaCl (S8776, Sigma‐Aldrich), and a stock solution of 40 U/mL thrombin in Tris‐buffered saline (PPB001, Sigma‐Aldrich). All solutions were thawed immediately before use and kept on ice. The fibrinogen/dPBS mixture was freshly prepared and rapidly mixed with thrombin by pipetting to achieve a final concentration of 10 mg/mL fibrinogen and 10.5 U/mL thrombin (605157‐1KU, Sigma‐Aldrich). The resulting fibrinogen–thrombin mixture was immediately combined with spheroid‐containing BM at a 1.35:1 ratio. The plates were incubated at 37°C for at least 20 min to allow complete gelation.

#### 2.1.8 Preparation of Fibrin‐Agarose–Gelatin (FibAgGel) Hydrogel and Spheroid Embedding

FibAgGel blend, which contained 0.35% (w/v) low‐melting agarose (A01690, Sigma‐Aldrich), 3% (w/v) gelatin (G1890‐500G, Sigma‐Aldrich), 5 mg/mL fibrinogen, and 5 U/mL thrombin, was prepared by mixing agarose and gelatin (both diluted in dPBS), vortexing the mixture gently and incubating it at 37°C to equilibrate. Freshly thawed fibrinogen was then added to the agarose–gelatin solution, followed by gentle pipetting to homogenize the hydrogel precursor. For embedding, 72% of the hydrogel precursor was pipetted into each well of a flat‐bottom 96‐well plate containing 28% of spheroid‐containing BM, and gelation was initiated by placing the plate at 4°C for 10 min. Fibrinogen polymerization was initiated by adding thrombin on top of the gel. The plate was incubated at 37°C for 20 min to allow complete gelation.

#### 2.1.9 Preparation of Collagen–Methylcellulose (CollMet) Hydrogel and Spheroid Embedding

2.5 mg/mL collagen type I (C3867‐1VL, Sigma‐Aldrich) was neutralized with ∼9% v/v ice‐cold 0.2 N NaOH (1091371000, Merck, Darmstadt, Germany) and 1x Medium 199 (M0650‐100ML, Sigma‐Aldrich), and mixed at a 1:1 ratio with spheroids in 1.2% methylcellulose (M0512‐100G, Sigma‐Aldrich, viscosity: 4000 cP), prepared as described in Tetzlaff et al., 2018 [23] in Endothelial Cell Basal Medium 2 (C‐22211, PromoCell). All components were maintained on ice prior to mixing to prevent premature gelation. Gels were polymerized by incubation at 37°C in a humidified incubator with 5% CO_2_ for 20–30 min.

#### 2.1.10 Fabrication of Planar Gel Layers with Evenly Distributed Spheroids

Negative silicone stamps were made using Aggrewell 800 plates as described above. A 6‐mm biopsy punch needle (I4100600, Mediware/Servoprax, Wesel, Germany) was used to cut out a stamp fitting into a well of a 96‐well plate. On the day of embedding, a negative silicone stamp was placed onto a thin layer of 5% gelatin in dPBS in a 96‐well plate, and the gels were incubated at 4°C for 15 min. After gelation, transglutaminase (B07B4TYFDJ, Wuerzteufel, Empfingen, Germany) in dPBS was added on top of the gelatin to a final concentration of 4 mg/mL, and the gels were incubated for 15 min at RT to achieve gelatin cross‐linking. Spheroids containing 16 000 cells each were loaded into the microwells in a minimal amount of medium. Fibrin gel was then mixed with the spheroids as described above (final volume of 60 µL) and carefully layered over the gelatin matrix, avoiding bubbles and ensuring proper spheroid positioning in the microwells. The plates were incubated at 37°C for at least 20 min to allow complete gelation. Finally, 200 µL of medium were added on top of the gel, and the medium was exchanged daily.

#### 2.1.11 Fabrication of Multilayer Tissue Constructs from Vascularized Spheroids

Planar tissue layers were fabricated on a Neoveil Mucosal Substitute sheet (Gunze International Europe GmbH, Düsseldorf, Germany) as described above and cultured for 7 days in 96‐well plates with daily medium changes. This sheet is a highly porous nonwoven polyglycolic acid membrane of 150 µm thickness, consisting of thin fibers and fiber bundles; based on light microscopy analyses, we determined an average distance of 713 µm between major fiber bundles. Three of such layers were stacked and kept in culture in 48 well‐plates with daily medium changes.

### 2.2 Staining of Spheroids

All antibodies used in the staining procedures are listed in Tables S1 and S2. Spheroids were fixed in 4% paraformaldehyde (PFA, 0335.3, Carl Roth) in dPBS for 1 h at RT with gentle rotation, washed twice with PBS, dehydrated, and paraffin‐embedded. The spheroids were cut into 5‐µm‐thick sections using a Jung RM2055 microtome (Leica Biosystems, Nußloch, Germany) and mounted on Epredia Polysine Adhesion microscope slides (J2800AMNZ, Microm International GmbH, Dreieich, Germany). Prior to stainings, sections were deparaffinized and rehydrated.

*For immunofluorescence staining*, sections were incubated in epitope retrieval citrate buffer (10 mM Sodium Citrate, 0.05% Tween‐20, pH 6.0) for 40 min in a water bath heated to 100°C and subsequently cooled for 40–60 min at room temperature (RT). After a washing step in PBS, sections were permeabilized in 0.5% Triton X‐100 in PBS for 10 min and blocked with 5% IgG‐free BSA (3737.1, Carl Roth, Karlsruhe, Germany) in PBS. Primary antibodies were applied in PBS containing 0.01% Triton X‐100 and incubated overnight at 4°C; PBS with 0.01% Triton X‐100 served as negative control. Sections were washed in PBS (3 × 10 min), followed by a 1‐h incubation step with respective secondary antibodies diluted in 0.01% Triton X‐100 in PBS. Specimens were then washed with PBS (2 × 10 min), counterstained with 4′,6‐diamidino‐2‐phenylindole (DAPI,1 µg/mL, 10236276001, Merck, Darmstadt, Germany) for 10 min, washed in PBS (5 min), rinsed in water, dehydrated in ethanol, and air‐dried. Mounting was performed with Fluorescence Mounting Medium (S3023, DAKO/Agilent, Waldbronn, Germany). Immunofluorescent images were acquired with Zeiss Observer D1 and Zeiss Axiovert 5 microscopes.

*For immunohistochemical staining*, antigen retrieval was performed in an EDTA‐containing buffer (FLEX kit, pH 9, Dako) in a steamer at 100°C for 30 min. Blocking, staining, and washing were performed in an autostainer (Thermo Scientific) using Dako EnVision FLEX reagents according to the manufacturer's protocols. Sections were mounted with Entellan (1079610500, VWR, Darmstadt, Germany).

*Periodic acid‐Schiff (PAS) staining* was performed using standard procedures on a Leica automated stainer. The deparaffinized sections were treated with periodic acid for 4 min, followed by incubation with Schiff's reagent for 6 min. Subsequently, the slides were washed in sulfur dioxide‐containing water for 4 min. Nuclei were counterstained with Gill's hematoxylin, after which the sections were dehydrated through a graded series of isopropanol, cleared in xylene, and mounted.

*For whole‐mount immunofluorescent staining of spheroids for confocal microscopy*, triple‐cell spheroids cultured for 14 days in EH 2%FCS or EH 4%SR as described above were washed twice with PBS and fixed in 4% PFA in PBS for 1 h at RT. Following fixation, spheroids were washed twice with PBS and stored at 4°C. The spheroids were blocked and permeabilized in PBS containing 1% BSA, 3% FCS, 0.5% Triton‐X‐100, 0.5% Tween‐20, 0.01% sodium deoxycholate overnight while shaking. The primary antibodies (anti‐VE‐cadherin and anti‐vimentin) diluted in antibody dilution buffer (blocking buffer diluted two times with PBS) were added, and spheroids were incubated over three nights at 4°C under gentle rotation to ensure homogeneous penetration throughout the entire 3D structure. Thereafter, the spheroids were incubated with staining solution three times 10 min each at RT with continuous gentle agitation. Fluorophore‐conjugated secondary antibodies diluted 1:300 were applied in antibody dilution buffer and incubated overnight at 4°C under gentle rotation, protected from light. The samples were then washed with staining solution three times for 5 min. Nuclear staining was performed with 1 µg/mL DAPI (6335.1, Carl Roth) in PBS for 15 min at RT. The samples were then washed with staining solution three times for 5 min. For optical clearing of whole spheroids, samples were incubated for several min with prewarmed RapiClear 1.52 (RC152001, Sunjin lab) at RT, until the spheroids appeared translucent. The spheroids were then transferred between two coverslips separated by iSpacers (IS009, Sunjin lab; 1 mm depth, 7 mm diameter). The samples were dried overnight at RT in the dark. Nail polish was used for sealing. Imaging was performed using a Carl Zeiss 980 inverted confocal scanning microscope, with a Zeiss Plan Apochromat 20x / 0.8 NA PH2 air‐objective. Z‐stacks covering the entire spheroid volume were acquired.

*To stain the spheroids embedded in hydrogels*, the gels were washed with PBS (3 × 1 min), and the spheroids in gel were fixed in 4% PFA in PBS for 30 min at RT, followed by three PBS washes. Permeabilization was done in 0.5% Triton X‐100 in PBS for 5 min at RT, followed by three washes with PBS. Blocking was done with 3% IgG‐free BSA in PBS for 1 h at RT. Primary antibodies were applied in PBS containing 3% IgG‐free BSA and incubated overnight at 4°C. Gels were washed three times with PBS, followed by a 1 h incubation with the appropriate secondary antibodies diluted in 3% IgG‐free BSA in PBS. Specimens were then washed once with PBS, incubated in 1 µg/mL DAPI for 10 min, washed again with PBS for 5 min, and kept in fresh PBS during imaging. Immunofluorescent images were taken on the same day with a Zeiss Observer D1 microscope.

*Staining of vascularized spheroids in microfluidic chips*. Four days after embedding into the chip, the cells were fixed with 4% PFA for 10 min at RT, followed by washing with 0.5% Triton X‐100 in PBS. The samples were blocked with blocking buffer (0.5% Triton X‐100, 0.01% sodium deoxycholate, 1% bovine serum albumin (A9418‐50G, Sigma‐Aldrich), 3% FCS) for 1 h at RT. The primary antibodies were diluted in a 1:1 mixture of blocking buffer and PBS and incubated for 3 days at 4°C with gentle shaking. After washing with PBS containing 0.5% Triton X‐100 (3 × 10 min), the chips were incubated overnight with secondary antibodies at 4°C on a shaker. After washing with PBS containing 0.5% Triton X‐100 (3 × 10 min), the chips were incubated with DAPI for 20 min at RT on a shaker, followed by another washing step with PBS. Images were obtained using a 3i spinning‐disc confocal microscope equipped with a Zeiss Plan‐Apochromat 20x/1.0 NA water‐dipping objective and SlideBook imaging software.

*Staining of multilayer tissue constructs*. Multilayer tissue was washed three times with PBS and fixed in 4% PFA in PBS for 30 min at RT, followed by three PBS washes, after which the stacked tissue was transferred into a 25% sucrose/PBS solution and incubated for 2 days at 4°C. On the following day, the tissue was embedded in Tissue‐Tek O.C.T. Compound (4583, Sakura Finetek, USA) and subsequently transferred onto dry ice for quick freezing and kept at ‐20°C until further use. Cryosections were generated using a Leica CM3050 Cryostat device. Sections of 30–40 µm thickness were cut and mounted on SuperFrost Plus Adhesion slides (J1800AMNZ, Epredia, Netherlands).

Cryosections of the tissue construct, approximately 0.8 and 1.8 mm from the outer edge, were rehydrated in PBS for 15 min at RT and subsequently permeabilized with 0.5% Triton X‐100 in PBS for 5 min at RT. Non‐specific binding was blocked using 3% IgG‐free BSA (3737.1, Carl Roth) in PBS for 30 min at RT in a humidified chamber. Primary antibodies were diluted in blocking solution and applied to the sections for overnight incubation at 4°C in a humidified chamber. Blocking solution without primary antibody served as negative control. The following day, slides were washed twice in PBS before incubation with secondary antibodies, diluted in blocking solution and precleared by centrifugation (3 min, 10 000 rpm). Secondary antibody incubation was performed for 1 h at RT in the dark. Slides were washed twice in PBS and counterstained with DAPI for 10 min at RT in the dark. After an additional 5‐min PBS rinse, sections were briefly washed in ultrapure water, followed by dehydration in 100% ethanol. Slides were air‐dried in the dark and mounted using Fluorescence Mounting Medium (S3023, DAKO/Agilent). Samples were imaged using a Zeiss Observer D1 microscope.

### 2.3 Transmission Electron Microscopy

Spheroids were fixed in 2.5% glutaraldehyde, stained in osmium tetroxide and embedded in Epon. Ultrathin sections were prepared for microscopy according to routine procedures. Electron microscopy was performed using a Jeol Jem 1400HC microscope (JEOL Ltd., Akishima, Tokyo, Japan) with a mounted 4k camera.

### 2.4 Image Analysis

The projected spheroid area was determined manually using ImageJ (35–69 spheroids per condition, three biological replicates). The Ki67‐positive and E‐cadherin‐positive cells were manually counted using the CellCounter plugin of the ImageJ software (https://imagej.net/ij/, National Institutes of Health, Bethesda, MD, USA). To determine the percentages of Ki67‐positive and of E‐cadherin‐positive cells, the number of the counted positive cells was divided by the total number of cells, as determined by DAPI staining. Ten spheroids per condition were analyzed.

The number of spheroids with sprouts in different hydrogels was analyzed in three independent experiments (three biological replicates with three technical replicates each (132–316 spheroids per condition)). In addition, the sprout length, defined as the linear distance from the spheroid center to the sprout tip, was measured in three independent experiments (three biological replicates with three technical replicates each), *n* (fibrin, EH 2%FCS) = 35, *n* (fibrin, EH 4%SR) = 22, *n* (FibAgGel, EH 2%FCS) = 8, *n* (FibAgGel, EH 4%SR) = 6, (CollMet, EH 2%FCS) = 17, *n* (CollMet, EH 4%SR) = 7. In the setup with defined spheroid positioning, the sprout length was measured for all sprouts emerging from the spheroids placed in nine wells. For 82 spheroid pairs, the inter‐spheroid distance, that was defined as the distance between the centers of two spheroids, was measured, and it was noted whether neither spheroid (0/2), only one spheroid (1/2) or both spheroids (2/2) exhibited sprouts. All measurements were performed using ImageJ.

The longest endothelial cell sprouts from each spheroid (determined as the linear distance from the spheroid center to the end of a sprout) in an evenly positioned 3 × 3 planar spheroid layer setup were measured in three independent experiments (three biological replicates, *n* (EH 2%FCS) = 38, *n* (EH 4%SR) = 53 spheroids).

For quantification of the number of spheroids forming anastomoses with the parent vessel in the microfluidic chip, only spheroids located 400–600 µm from the parent vessel were included in the analysis. At distances greater than 600 µm, spheroid‐derived HUVECs preferentially formed sprouts toward the opposite, pericyte‐containing channel. At distances shorter than 400 µm, RFP‐labeled HUVECs from the parent vessel did not extend sprouts toward the center of the chip. Within this permissive range, spheroids were categorized and counted as follows: (i) spheroids forming anastomoses with the parent vessel, (ii) spheroids forming sprouts toward the parent channel without establishing an anastomosis within the 5‐day experimental period, and (iii) spheroids showing no connection to the parent vessel. In total, five chips with 14 spheroids and 7 chips with 26 spheroids were analyzed for EH 2%FCS and EH 4%SR, respectively.

### 2.5 3D Visualization, Segmentation, and Quantitative Analysis of the Confocal Images

3D visualization, segmentation, and quantitative analysis of the confocal images of the triple‐cell spheroids stained with anti‐VE‐cadherin antibodies were performed using Imaris (Oxford Instruments, v11). Endothelial (VE‐cadherin‐positive) cells and spheroids were segmented using the Surface module. Spheroids were segmented manually by drawing contours at 10‐µm intervals along the *z*‐axis. Automated surface detection of VE‐cadherin‐positive structures was performed on the VE‐cadherin fluorescence channel with a surface detail level of 1.5 µm, determined based on manual measurements of the smallest features. Background subtraction was applied using the local contrast method. Intensity thresholds for surface detection were applied uniformly across all samples. A minimum voxel count threshold of 2000 voxels was then used to exclude debris. The resulting surfaces were masked and used as input for filament reconstruction using the Filament module. Vascular networks were reconstructed using the Autopath algorithm (loops enabled; no Soma and no Spine) with seed point diameters ranging from 1, 5 to 9 µm. Filament reconstructions were visually inspected for accuracy, and datasets with obvious tracing artifacts were excluded from further analysis. The reconstructed filament networks were used for automated quantification of branch points, total vessel length, and mean diameter of each vessel segment. The average of mean diameter values was calculated for each spheroid, and subsequently the group mean ± SD was determined. In addition, maximal vessel diameter was determined for each spheroid. The vessel fraction within spheroids was calculated as the ratio of vessel volume to the total spheroid volume, as determined using the Surface module. All analyses were performed using identical parameters across samples.

### 2.6 Perfusion

PBS was removed from all four wells of the fixed microfluidic chips. The chips were then incubated in an upright position with 1 µm fluorescent beads (F8816, Invitrogen, FluoSpheres carboxylate‐modified microspheres (625/645)) diluted 1: 10000 in PBS for 2 h under gentle shaking to allow the beads to settle into the vessels. Fluorescence imaging was performed using a Leica Mica Microhub (Leica Microsystems, Wetzlar, Germany). Images were acquired using Leica N PLAN 10x/0.25 air objective and visualized with LAS X software.

### 2.7 Albumin Enzyme‐Linked Immunosorbent Assay (ELISA)

Albumin secretion was quantified using the Human Albumin ELISA Kit (E88‐129, Bethyl Laboratories, Montgomery, Texas, USA) according to the manufacturer's instructions. Briefly, HepaRG monoculture spheroids or triple‐cell spheroids were generated and cultured as described above. 1.2 × 10^6^ total cells including a 5/9 fraction (5:2:2 ratio) of HepaRG cells in triple‐cell spheroids or 1.2 × 10^6^ of HepaRG cells in case of monoculture spheroids were used per well to generate 300 spheroids, and supernatant from one well was collected. Prior to supernatant collection, the indicated medium was exchanged on days 10 and 17, and the cell culture supernatants were collected on days 14 and 21, respectively. This collection scheme recapitulates the applied culture conditions. The supernatants were diluted 1:200 in 1X Dilution Buffer C. Albumin standards were prepared by serial dilution to generate a calibration curve. Standards and samples were added to antibody‐precoated 96‐well plates and incubated for 1 h at RT. After four washing steps with the provided Wash Buffer, anti‐albumin Detection Antibody was applied and incubated for 1 h at RT, followed by four additional washes. For enzyme labeling, HRP Solution A was added for 30 min. Following four washing steps with Wash Buffer, the signal was developed using tetramethylbenzidine substrate, and the reaction was stopped with Stop Solution. Absorbance was measured at 450 nm using a Tecan Spark multimode microplate reader (Tecan Group Ltd., Maennedorf, Switzerland). Albumin concentrations were calculated based on a four‐parameter logistic (4PL) standard curve using GraphPad Prism 8 (GraphPad software San Diego, CA) and normalized to one spheroid. Three biological replicates were analyzed per sample.

### 2.8 Urea Measurements

Urea concentrations in cell culture supernatants were measured using the QuantiChrom Urea Assay Kit (DIUR‐100, BioAssay Systems, Hayward, California, USA) following the manufacturer's instructions. HepaRG monoculture spheroids or triple‐cell spheroids were generated and cultured as described above. 1.2 × 10^6^ total cells including a 5/9 fraction (5:2:2 ratio) of HepaRG cells in triple‐cell spheroids or 1.2 × 10^6^ of HepaRG cells in case of monoculture spheroids were used per well to generate 300 spheroids, and supernatant from one well was collected. Prior to supernatant collection, the indicated medium was exchanged on days 10 and 17, and the cell culture supernatants were collected on days 14 and 21, respectively. This collection scheme recapitulates the applied culture conditions. 50 µL of undiluted supernatant or standard (5 mg urea/dL) were added to a 96‐well plate, followed by 200 µL of the working reagent provided with the kit. Plates were incubated at RT for 20 min, allowing for color development. Absorbance was measured at 520 nm using a Tecan Spark multimode microplate reader. Urea concentrations were calculated based on a standard curve generated with known urea standards and normalized to one spheroid. Three biological replicates were analyzed per sample.

### 2.9 Cytochrome P450 family 3 subfamily A member 4 (CYP3A4) Activity Assay

HepaRG monoculture spheroids or triple‐cell spheroids were generated and cultured as described above and used at days 14 or 21 of culture. CYP3A4 activity was quantified using a luminescence‐based P450‐Glo CYP3A4 Assay with Luciferin‐IPA for cell‐based samples (V9001, Promega Corporation, Madison, Wisconsin, USA) following the manufacturer's protocol with minor modifications. For CYP3A4 induction, spheroids were treated with 50 µM dexamethasone for 24 h prior to the assay. Spheroids were collected into a U‐shaped 96‐well plate and incubated in 100 µL of corresponding medium (EH 2%FCS or EH4 %SR) containing the luminogenic substrate luciferin‐IPA (3 µM final concentration) for 60 min at 37°C, 5% CO_2_ while shaking. Following incubation, 50 µL of supernatant was transferred from each well to a white opaque 96‐well plate. An equal volume (50 µL) of reconstituted Luciferin Detection Reagent was added, and the plate was incubated with gentle shaking for 20 min at RT to stabilize the luciferase reaction. Luminescence was measured using a Tecan Spark multimode microplate reader with 1 s integration time per well. Background luminescence (corresponding medium + substrate + detection reagent without spheroids) was subtracted from all samples. CYP3A4 activity was expressed as relative light units (RLU) per well with 300 spheroids formed by 1.2 × 10^6^ cells. Measurements were performed with at least three independent biological replicates.

### 2.10 Grafting Triple‐Cell Human Liver Spheroids into Mouse Liver Explants

All animal procedures were performed in compliance with German national guidelines and approved by the local authorities (approval for the conduct of scientific experiments DA8/2000, Regierungspräsidium Darmstadt, Hesse, Germany). *Smarcb1^+/inv^ NesCre^−/−^* P0 mice [24] were sacrificed for the purpose of another study (DA8/2000), and the remaining liver tissue was used for this study. Following isolation, the mouse liver explants were immediately placed into EH 2%FCS medium and incubated at 37°C in a humidified 5% CO_2_ incubator. After a 2‐h recovery phase, 4‐days‐old triple‐cell liver spheroids, generated according to the protocol above, were grafted into mouse liver explants using a 30G 0.30 × 12 mm syringe (22834, Braun, Melsungen, Germany). The grafted liver explants were incubated in EH 2%FCS medium and imaged on days 1 and 2 after injection using a Zeiss Observer D1 microscope.

### 2.11 Compression Test

Compression tests were executed in triplicates for FibAgGel and fibrin gels. CollMet samples could not be measured as the cross‐linked state was too liquid for this kind of measurement. The gels were casted into agarose wells with a diameter of 1.1 cm, left to gelate and afterward warmed to 37°C before placed into the rheometer. The rheometer was warmed to 37°C, the final gap was set to 0.2 mm, and the travel speed to 0.1 mm/s. The casted gels were dome‐shaped rather than cylindrical, which should be taken into consideration as the calculations are based on the assumption that the gels are cylindrical and have a diameter of 1.1 cm. The Tangent Young's Modulus was calculated for the first 1.5 mm of compression, based on the following formula, where m is determined by the slope of the corresponding range in the force‐displacement diagram: *E* = *L*_0_/A*m. Three samples were measured to determine the mean value and standard deviation.

### 2.12 RNA Isolation

Approximately 300 spheroids were used for RNA isolation using the *Quick*‐RNA Microprep Kit (R1050, Zymo Research Europe GmbH, Freiburg i.Br., Germany) according to manufacturer's instructions and eluted in 15 µL nuclease‐free water.

### 2.13 Quantitative Reverse Transcription Polymerase Chain Reaction (RT‐qPCR)

To synthesize complementary DNA (cDNA), 1 µg RNA diluted in nuclease‐free water to a volume of 12 µL were mixed with 1 µL Random Hexamer Primers (SO142, Thermo Scientific) and incubated for 5 min at 65°C. Reaction buffer, 10 mM dNTPs, 20 U Ribolock (EO0381, Thermo Scientific), and 1 µL Revert Ad H Minus M‐MuLV Transcriptase (EP0451, Thermo Scientific) were added. Samples were incubated at RT for 10 min, at 42°C for 60 min, and reverse transcriptase was inactivated at 70°C for 10 min. The resulting cDNA was diluted 1:100 prior to amplification. qPCR reactions were prepared in a final volume of 25 µL containing 12.5 µL PowerUp SYBR Green Master Mix (A25743, Applied Biosystems, Thermo Fisher Scientific Baltics UAB, Vilnius, Lithuania), 0.5 µL each of 10 mM gene‐specific forward and reverse primers, nuclease‐free water, and 4 µL cDNA template. Amplification was carried out on a StepOne thermal cycler under the following cycling conditions: polymerase activation at 95°C for 10 min, followed by 40 cycles of denaturation at 95°C for 15 s and annealing/extension at 58°C for 1 min. Melt curve analysis was performed at the end of each run to verify amplification specificity. Relative gene expression levels were calculated using the ΔΔCt method after normalization to the housekeeping genes *YWHAZ* and *TBP*. The reference genes were selected using RefFinder [25]. Three biological replicates with three technical replicates were used per sample. The primers used are listed in Table S3.

### 2.14 Statistical Analysis

GraphPad Prism 8 was used for statistical tests and graphical display. Normality of data within groups was assessed using the Shapiro–Wilk test. For normally distributed data, group comparisons were performed using the Games–Howell's multiple comparisons test, which accounts for unequal variances and sample sizes, to determine whether the following datasets were significantly different: projected spheroids area; percentages of Ki‐67‐positive and E‐cadherin‐positive cells; percentages of triple‐cell spheroids with sprouts cultured in EH 2%FCS or EH 4%SR after embedding in indicated hydrogels, sprout lengths for the spheroids described above, and maximal sprout length in triple‐cell liver spheroids cultured in EH 2%FCS or EH 4%SR on day 3 after embedding in the indicated hydrogels. Significance levels were defined as n.s. (not significant, *p* > 0.05), *p ≤ 0.05, **p ≤ 0.01, ***p ≤ 0.001. Data are presented as means ± SEM. Datasets describing whether neither spheroid (0/2), only one spheroid (1/2), or both spheroids (2/2) in spheroid pairs with defined distances exhibited sprouts were not all normally distributed; thus, the Kruskal–Wallis test was applied. The longest sprout lengths of endothelial cells from positioned triple‐cell spheroids cultured in EH 2%FCS or EH 4%SR were compared using an unpaired *t*‐test with Welch's correction. The compression test data were compared using an unpaired *t*‐test with Welch's correction.

For RT‐qPCR data and functional assays, the Shapiro–Wilk test was performed to assess whether the data were normally distributed. If the data were normally distributed, unpaired *t*‐test with Welch's correction was applied to test whether the datasets are significantly different. If the data were not normally distributed, Mann–Whitney test was used. Data are presented as means ± SEM. n.s. not significant (*p* > 0.05), *p ≤ 0.05, ** *p* ≤ 0.01, ***p ≤ 0.001.

## 3 Results

### 3.1 Mesenchymal Stem Cells Facilitate Endothelial Cell Network Formation Within Human Liver Cell Spheroids

The HepaRG cell line is a widely used human liver cell line for research, drug development, and toxicology assessment [26, 27]. HepaRG cells can be expanded and maintained in culture for extended periods while retaining stable hepatic functions and phenotype, and can differentiate into two distinct liver cell types: hepatocyte‐like cells and cholangiocyte‐like cells. Endothelial cells such as HUVECs, a well‐established and easily accessible primary endothelial cell model from the vein of the human umbilical cord, possess the inherent capacity to self‐organize into tube‐like structures in vitro when placed onto or within extracellular matrix proteins or hydrogel mimics thereof [28, 29].

Since HepaRG cells are principally capable of producing extracellular matrix proteins, [30] we set out to test if HUVECs could form vascular network‐like structures in 3D HepaRG spheroids upon aggregation of single cells (Figure 1). HUVECs stably expressing GFP were used to monitor their position within spheroids by fluorescence microscopy. Single‐cell suspensions with HepaRG to GFP‐HUVEC cell ratios of 7:2, 5:2, or 5:4 in a medium optimized for endothelial cells, Endothelial Cell Growth Medium 2 containing 2% FCS (ECGM2 2%FCS), were transferred into microwell plates to generate spheroids containing 4000 cells each. On day four, formed spheroids were transferred to suspension plates and placed on a shaker (Figure 1A). In case of all three HepaRG to GFP‐HUVEC cell ratios, the two cell types separated over the course of 6 days, with HUVECs finally located in the spheroid center surrounded by non‐fluorescent HepaRG cells (Figure 1B). Based on these results, we decided to add human mesenchymal stem cells (MSCs) from adipose tissue that are known to produce abundant extracellular matrix, are present in the stromal vascular fraction, and resemble pericytes [31]. These cells are highly accessible and provide a rich and practical source for regenerative medicine and research applications. Triple‐cell spheroids composed of HepaRG, GFP‐HUVEC, and MSC cells at varying ratios were cultured for an initial observation period of 6 days, and green fluorescent signals were monitored (Figure 1C). These experiments revealed that MSCs supported the formation of network‐like endothelial structures in case of the following ratios: HepaRG: GFP‐HUVEC: MSC 5: 2: 2 and 5: 2: 4 (Figure 1C, see the fluorescence microscopy images from day 6 in the bottom row, third and fourth images from the left). A lower GFP‐HUVEC or MSC content (HepaRG: GFP‐HUVEC: MSC ratios 5: 1: 2 and 5: 2: 0.5) or higher proportion of GFP‐HUVECs within the spheroids (HepaRG: GFP‐HUVEC: MSC ratio 5: 5: 2) resulted in segregation of GFP‐HUVECs and absence of network‐like endothelial structures (Figure 1C, see the fluorescence microscopy images from day 6 in the bottom row). Based on these observations, subsequent experiments employed spheroids generated at a cell ratio of 5:2:2 (HepaRG: GFP‐HUVEC: MSC), aiming to maximize the fraction of liver cells while maintaining vascular network formation.

### 3.2 Vascularized Liver Spheroid Growth is Influenced by Cell Culture Medium Composition

**FIGURE 2:**
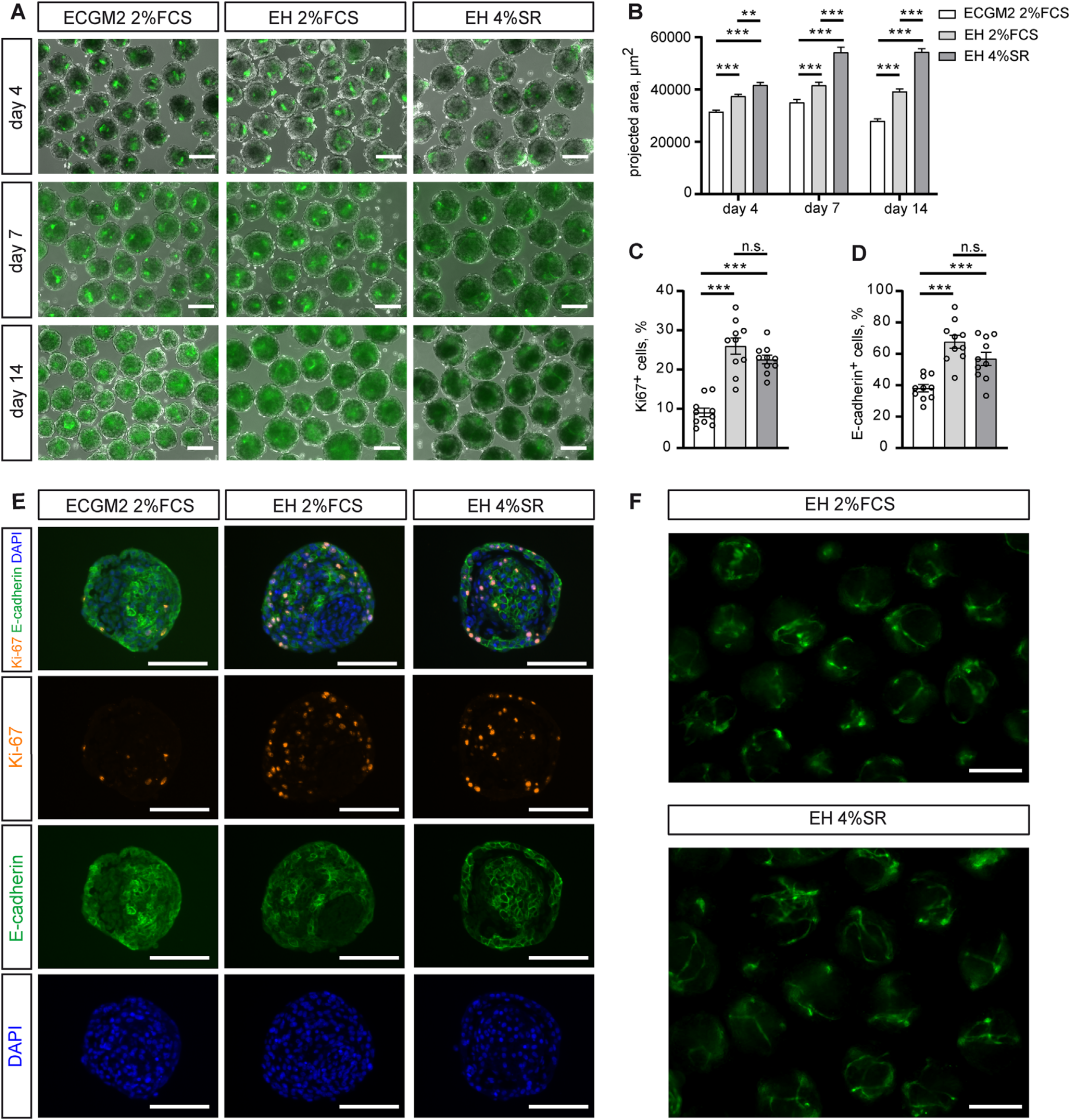
Comparison of three different media compositions (ECGM2 2%FCS, EH 2%FCS, and EH 4%SR). The serum‐reduced and serum‐free media EH 2%FCS and EH 4%SR support triple‐cell liver spheroid growth and endothelial cell networks. (A) Merged bright‐field and GFP fluorescence microscopy images of spheroids composed of HepaRG cells, GFP‐HUVECs, and MSCs at a ratio of 5:2:2 that were cultured in ECGM2 2%FCS, EH 2%FCS, or EH 4%SR for 4, 7, or 14 days. (B) Projected area of spheroids cultured in ECGM2 2%FCS, EH 2%FCS, or EH 4%SR on days 4, 7, and 14 after cell seeding. A graphical representation with individual data points is shown in Figure S1. *n* = 35–69 spheroids per condition, three biological replicates. (C, D) Numbers of Ki‐67‐positive (C) and E‐cadherin‐positive (D) cells within spheroids cultured in ECGM2 2%FCS, EH 2%FCS, or EH 4%SR on day 7 after seeding. Ten spheroids per condition were analyzed. (E) Representative Ki‐67 and E‐cadherin immunofluorescence images of paraffin sections of spheroids cultured in ECGM2 2%FCS, EH 2%FCS, or EH 4%SR on day 7 after cell seeding. 4′,6‐diamidino‐2‐phenylindole (DAPI) staining marks the cell nuclei. (F) Representative fluorescence microscopy images showing GFP‐positive network structures within spheroids cultured in EH 2%FCS or EH 4%SR for 7 days. (A, E, F): Scale bars: 100 µm. (B, C, D): Means ± SEM are shown. The Games–Howell's multiple comparisons test, which accounts for unequal variances and sample sizes, was applied to determine whether the datasets were significantly different. *p ≤ 0.05, **p ≤ 0.01, ***p ≤ 0.001, n.s.—non‐significant. (C,D): individual data points are indicated.

Although the ECGM2 2%FCS medium supported the formation of vascular‐like structures within the spheroids, the spheroid size decreased over time (Figure 2A,B and Figure S1), indicating a long‐term growth impairment under these conditions. To better support HepaRG cells, we adapted the medium composition. In addition to 50% ECGM2 medium—including growth factors supporting endothelial cells at concentrations equivalent to 100% ECGM2 (5 ng/mL EGF, 10 ng/mL bFGF, 20 ng/mL IGF‐1 LR3, 0.5 ng/mL VEGF165, 1 µg/mL ascorbic acid, 22.5 µg/mL heparin, 0.2 µg/mL hydrocortisone)—we added 50% of William's E medium and 5 µg/mL insulin. This resulting medium was designated EH 2%FCS (Endothelial cell Hepatocyte medium with 2% FCS) to indicate its application for both endothelial cells and hepatocytes. These two cell types, as well as MSCs, could be expanded as adherent cells in EH 2%FCS (Figure S2). Spheroids cultured in EH 2%FCS were larger (Figure 2B and Figure S1), contained more Ki67‐positive, proliferating cells, and higher numbers of E‐cadherin‐positive HepaRG cells compared with the spheroids kept in ECGM2 2%FCS (Figure 2C,D,E). Importantly, the spheroids cultured in EH 2%FCS still retained extensive vascular‐like networks (Figure 2F).

We next aimed to identify a serum‐free medium with a defined composition that would support the growth of vascularized liver spheroids similar to the FCS‐containing medium. The exclusion of FCS eliminates the batch‐to‐batch variability inherent in animal serum, reduces the risk of introducing pathogens, aligns with increasing regulatory demands for defined, animal‐free media in cell‐based therapeutics, and supports the 3Rs principle in research. To this end, we replaced FCS with KnockOut Serum Replacement (SR) in the EH formulation, resulting in EH 4%SR. SR is a chemically defined, serum‐free medium supplement widely used to support embryonic stem cell and induced pluripotent stem cell growth without the variability associated with serum [32]. It can also support directed induced pluripotent stem cell differentiation and improve survival and yield of desired lineages [33, 34, 35]. SR contains basic nutrients such as amino acids, vitamins, antioxidants, and trace elements as well as key proteins, namely insulin, transferrin, and lipid‐rich albumin. Together, these components support metabolism, redox balance, and growth factor signaling. Insulin and transferrin stimulate cell proliferation and metabolism; albumin is a multifunctional carrier and stabilizer, transporting nutrients and growth factors while protecting cells from toxins and oxidative stress; thiamine, glutathione, and ascorbate support metabolic pathways and protect against stress. We reasoned that these functions would sustain hepatic and endothelial cells in vitro. Indeed, the spheroids cultured in EH 4%SR had the largest projected area in comparison to those kept in ECGM2 2%FCS and EH 2%FCS (Figure 2A,B and Figure S1) and displayed proliferating cells (Figure 2C,E), E‐cadherin‐positive HepaRG cells (Figure 2D,E), and vascular‐like structures (Figure 2F) comparable to the spheroids kept in EH 2%FCS. Importantly, under the EH 2%FCS and EH 4%SR culture conditions, the fraction of E‐cadherin‐positive hepatic and proliferating cells was significantly higher than under the ECGM2 2%FCS condition (Figure 2C,D). The high proportion of E‐cadherin‐positive hepatic cells in vascularized spheroids is a relevant achievement (67.66% ± 4.13% in EH 2%FCS; 56.79% ± 4.26% in EH 4%SR, means ± SEM, Figure 2D). Immunoreactivity for cleaved caspase‐3, an apoptosis marker, was not detected in spheroid sections under any of the tested cell culture conditions. The presented results indicate that the replacement of FCS with SR did not impair spheroid growth, cellular proliferation, or maintenance of hepatic and endothelial cell populations. Notably, the presence of vascular‐like structures in both conditions suggests that the SR‐containing medium supports endothelial organization similarly to the medium with FCS. These findings demonstrate that EH medium supplemented with 4% SR is a viable alternative to traditional FCS‐containing media for maintaining the structural and functional integrity of spheroids containing endothelial cells in combination with other cell types.

### 3.3 Vascularized Liver Spheroids Exhibit Characteristics of Human Liver Tissue and can be Maintained in Culture for 3 Weeks

**FIGURE 3:**
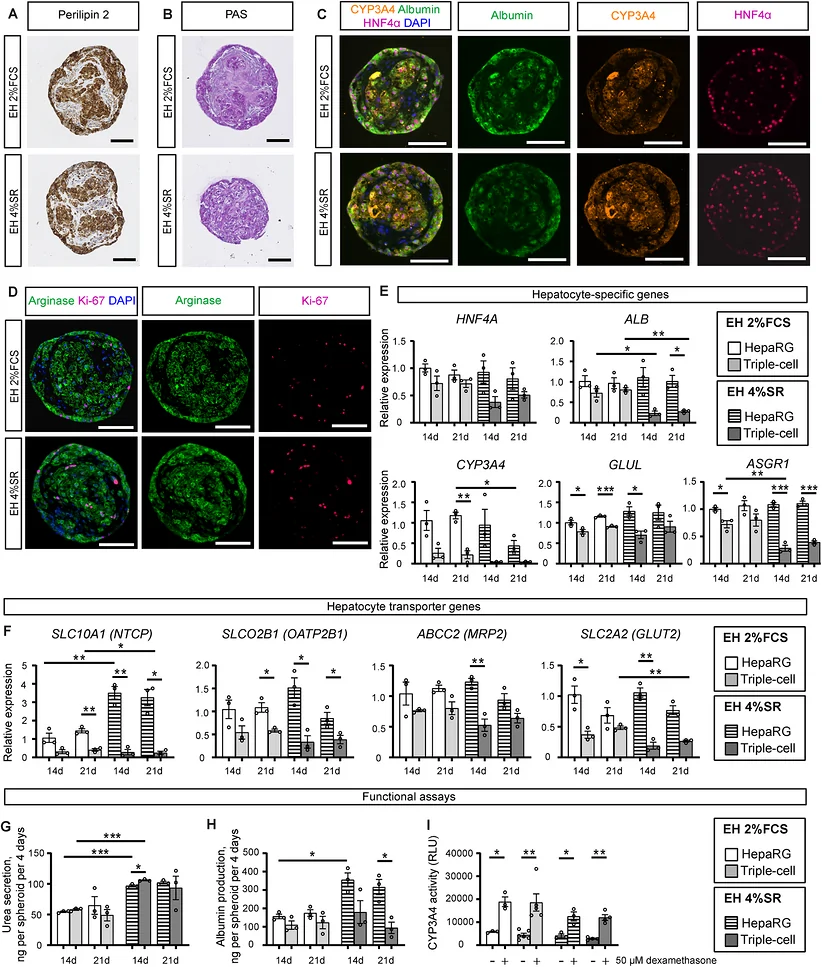
Hepatocyte and liver tissue features of triple‐cell liver spheroids based on PAS reaction of paraffin sections, immunostaining, RT‐qPCR, and functional assays. (A) Immunohistochemistry staining with anti‐perilipin 2 antibodies. (B) Periodic acid Schiff (PAS) reaction indicating the presence of glycogen and other carbohydrate‐containing entities. (C) Immunostainings with antibodies against albumin (green), CYP3A4 (orange), and HNF4α (magenta). DAPI staining is shown in blue. (D) Immunosignals depicting the hepatocyte marker arginase (green), and the proliferation marker Ki‐67 (magenta). DAPI staining is shown in blue. (A–D) Triple‐cell liver spheroids cultured in EH 2%FCS or EH 4%SR for 21 days. Scale bars: 100 µm. (E, F) Gene expression levels of hepatocyte‐specific genes *HNF4A*, *ALB*, *CYP3A4*, *GLUL*, and *ASGR1* (E), and hepatic transporters *SLC10A1 (NTCP), SLCO2B1 (OATP2B1), ABCC2 (MRP2)*, and *SLC2A2(GLUT2)* (F) in HepaRG monoculture spheroids or triple‐cell liver spheroids cultured in EH 2%FCS or EH 4%SR for 14 or 21 days. Data are presented as means ± SEM; three biological replicates are shown. (G, H) Human urea (G) and albumin (H) levels in the culture media of HepaRG monoculture spheroids or triple‐cell liver spheroids cultured in EH 2%FCS or EH 4%SR, normalized per spheroid. Cell culture supernatants were collected at the indicated time points. (I) CYP3A4 enzymatic activity (basal and induced by incubation with 50 µM dexamethasone for 24 h) in HepaRG monoculture spheroids or triple‐cell liver spheroids cultured in EH 2%FCS or EH 4%SR for 14 days. For (G–I), data are presented as means ± SEM; three biological replicates are shown. (E‐I) A Shapiro–Wilk test was performed to assess whether the data were normally distributed. If the data were normally distributed, an unpaired *t*‐test with Welch's correction was applied to test whether the datasets were significantly different. If the data were not normally distributed, a Mann–Whitney test was used. n.s. not significant (*p* > 0.05), *p ≤ 0.05, **p ≤ 0.01, ***p ≤ 0.001.

HepaRG cells are bipotent and can give rise to cholangiocytes and hepatocytes with the latter known to display different stages of maturity under different culture conditions [26, 35]. To assess the identity and distribution of liver cell types in the triple‐cell spheroids and to evaluate long‐term cultures, we kept spheroids in EH 2%FCS or EH 4%SR for 14 or 21 days (Figure 3 and Figure S3). Immunohistochemistry of paraffin sections demonstrated the presence of PAS‐positive hepatoid cell trabeculae containing many small lipid droplets (as evidenced by perilipin 2 positivity) in spheroids kept in both media for 21 days, consistent with a metabolically active, relatively mature hepatocytic phenotype (Figure 3A,B).

Immunostainings revealed expression of a master regulator of hepatocyte differentiation, hepatocyte nuclear factor 4α (HNF4α), along with key hepatocyte maturity markers including cytochrome P450 family 3 subfamily A member 4 (CYP3A4), albumin, and arginase, as well as the presence of proliferating liver cells (Figure 3C,D and Figure S3A,B). On the transcript level, among analyzed hepatocyte‐specific genes (*HNF4A*, *ALB*, *CYP3A4*, *GLUL*, and *ASGR1)* in triple‐cell liver spheroids, only *ALB* and *ASGR1* expression of day 14 spheroids and *ALB* level of day 21 spheroids differed between EH 2%FCS compared with EH 4%SR culture conditions (Figure 3E). In case of genes encoding hepatic transporters, significantly higher expression levels in triple‐cell liver spheroids cultured in EH 2%FCS were detected for *SLC2A2 (GLUT2)* on day 21 (Figure 3F). Importantly, there was no significant decrease in transcript levels of hepatocyte‐specific genes or hepatic transporters *SLC10A1* (*NTCP)*, *SLCO2B1* (*OATP2B1)*, *ABCC2 (MRP2)*, and *SLC2A2 (GLUT2)* with either of the two media between days 14 and 21 (Figure 3E,F). Compared with monoculture spheroids composed of HepaRG cells only and cultured in EH 2%FCS or EH 4%SR, the expression of several genes was lower in triple‐cell liver spheroids (Figure 3E,F). For all RT‐qPCR and functional assay values (see below), the lower proportion of hepatocytes in triple‐cell liver spheroids must be taken into account (mean 57%–68%, see Figure 2D).

To functionally assess the generated spheroids, we measured urea secretion, albumin production, and CYP3A4 activity of spheroids cultured in EH 2%FCS or EH 4%SR and compared these to test results obtained with non‐vascularized HepaRG monoculture spheroids (Figure 3G–I). Overall, there were no differences in urea secretion levels between triple‑cell and monoculture spheroids; a significant difference was only detected in case of spheroids cultured in EH 4%SR for 14 days (Figure 3G). Urea levels were generally higher in the EH 4%SR medium, reaching significance at day 14 (Figure 3G). Albumin production was comparable throughout different culture conditions and incubation days and differed only between HepaRG monoculture spheroids and triple‐cell spheroids under one condition: HepaRG monoculture spheroids showed a higher albumin production in comparison to EH 4%SR triple‐cell spheroids on day 21 (Figure 3H). CYP3A4 assays showed that the basal level of CYP3A4 activity could be further induced by 50 µM dexamethasone, and there was no difference between triple‐cell liver spheroids and HepaRG monoculture spheroids (Figure 3I).

**FIGURE 4:**
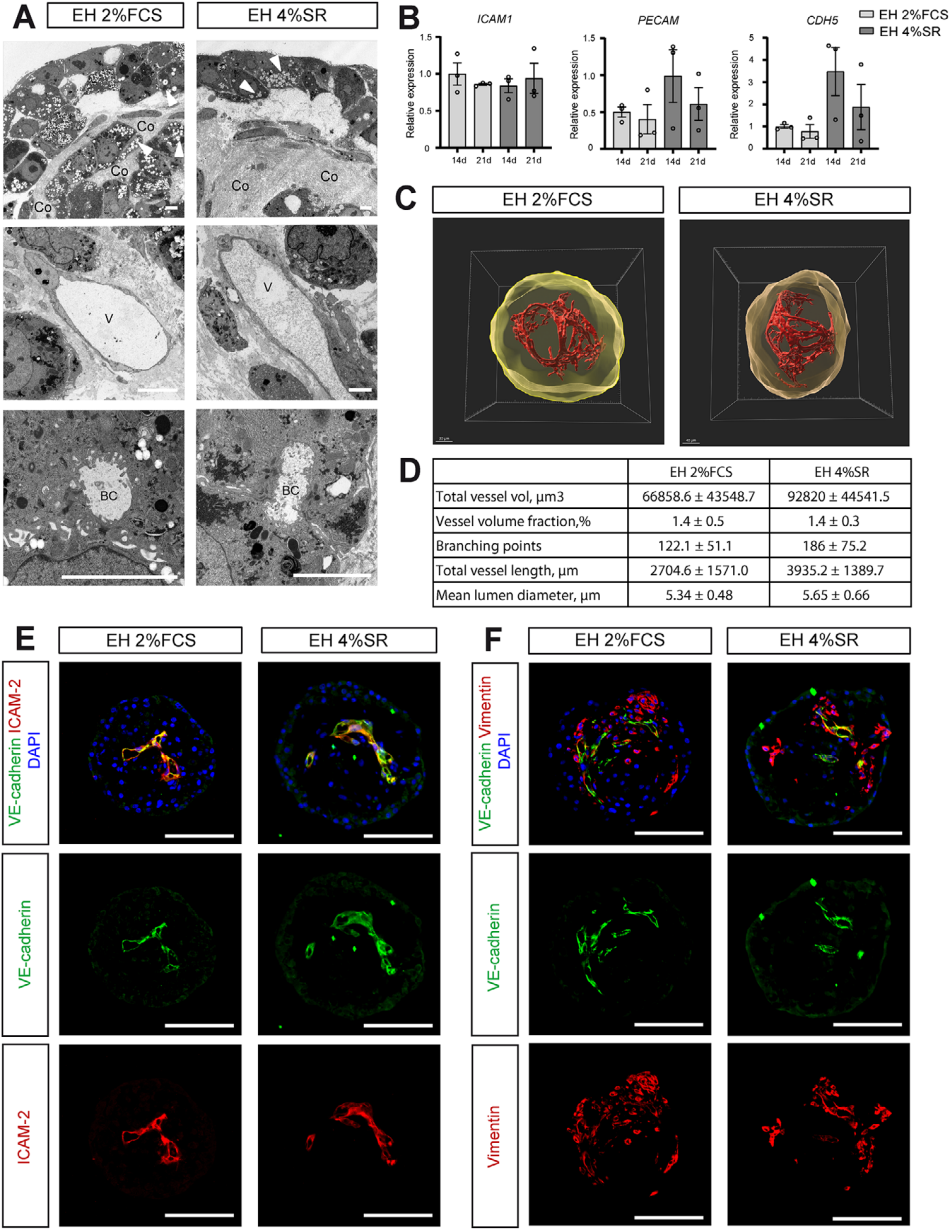
Characterization of vascular structures in the triple‐cell liver spheroids based on immunostainings, RT‐qPCR, 3D visualization and quantitative analysis of confocal images, and transmission electron microscopy. (A) Transmission electron microscopy of tissue sections from spheroids cultured in EH 2%FCS or EH 4%SR for 21 days. Upper row: hepatocytic cells at the outer region of spheroids with lipid droplets (white arrowheads) and collagenous stroma (indicated as “Co”). Middle row: capillary‐type vessels (V). Bottom row: bile canaliculi (BC) formation in hepatocytic cells. Scale bars: 5 µm. (B) Gene expression levels of endothelial markers *ICAM1*, *PECAM*, and *CDH5* in the triple‐cell liver spheroids cultured in EH 2%FCS or EH 4%SR for 14 or 21 days. Data are presented as means ± SEM; three biological replicates are shown. Unpaired *t*‐test with Welch's correction was applied to test whether the datasets are significantly different. (C) 3D reconstruction of confocal microscopy images of whole‐mount spheroids cultured in EH 2%FCS or EH 4%SR for 14 days and immunostained with anti‐VE‐cadherin antibodies. The VE‐cadherin positive vascular structures are shown in red. Scale bars: 100 µm. (D) Quantitative analysis of the vascular structures visualized by confocal microscopy of whole‐mount triple‐cell liver spheroids cultured in EH 2%FCS or EH 4%SR for 14 days and stained with an anti‐VE‐cadherin antibody. Means ± SD are shown. (E, F) Vascular‐like structures in spheroids cultured in EH 2%FCS or EH 4%SR for 21 days. Immunosignals of endothelial cell markers VE‐cadherin (E, F, green) and ICAM‐2 (E, red) and mesenchymal cell marker vimentin (F, red) in spheroid paraffin sections. DAPI staining is shown in blue. Scale bars: 100 µm.

Ultrastructural analyses of day 14 (Figures S4A and S5) and day 21 (Figure 4A and Figure S5) spheroids kept in both media revealed hepatocytic cells with a polygonal shape and well‐formed bile canaliculi, typical oval cell nuclei, lipid droplets, and glycogen accumulation. In addition, vascular structures embedded in a collagenous matrix were present. The compactness and polygonal shape of hepatocytes in spheroids at days 14 and 21 were similar; yet, more collagen fibrils appeared to be present in spheroids kept in EH 4%SR.

### 3.4 Endothelial Cells form Structures Resembling Microvessels Within Liver Spheroids

Transmission electron microscopy of spheroids revealed morphological features that are typical of capillaries: flat endothelial cells interconnected by plaque‐bearing adherens junctions in both media on days 14 (Figures S4A) and 21 (Figure 4A). RT‐qPCR analyses showed the expression of genes encoding endothelial transporters *ICAM1*, *PECAM1*, and *CDH5* in spheroids cultured in both media for 14 or 21 days (Figure 4B).

Confocal imaging of whole‐mount spheroids cultured in EH 2%FCS or EH 4%SR for 14 days and stained with VE‐cadherin and vimentin antibodies (Videos S1 and S2) demonstrated an interconnected vascular system within the spheroids. Obtained images were applied for 3D visualization, segmentation, and quantitative analysis of vascular structures (Figure 4C,D). Figure 4C shows VE‐cadherin‐positive vessels (red) forming an extensive meshwork within the spheroid core. Vessels exhibited hierarchical branching with multiple loops and anastomoses (Figure 4C), with a total vessel volume of 66 858.6 ± 43 548.7 µm^3^ and 92 820 ± 44 541.5 µm^3^, corresponding to a vessel volume fraction of about 1.4% for both types of spheroids (Figure 4D). The estimated total vessel length was 2704.6 ± 1571.0 µm for EH 2%FCS and 3935.2 ± 1389.7 µm for EH 4%SR (Figure 4D). The vascular structures in spheroids cultured in EH 2%FCS and EH 4%SR had 122.1 ± 51.1and 186 ± 75.2 branching points, respectively (Figure 4D). The mean lumen diameter was similar in both types of spheroids (5.34 ± 0.48 µm for EH 2%FCS and 5.65 ± 0.66 µm for EH 4%SR) (Figure 4D) and corresponded to the size of capillary vessels. The maximal lumen diameter reached 11.1 and 11.0 µm for EH 2%FCS and EH 4%SR, respectively.

HUVECs in the triple‐cell liver spheroids formed VE‐Cadherin‐ and ICAM‐2‐positive network‐like structures. Lumina within some of these structures were detectable on day 14 (Figure S4B) and on day 21 (Figure 4E and Figure S6A). In particular, the immunoreactivity of ICAM‐2 itself, a marker for apical/luminal endothelial cell membranes, is consistent with the presence of lumina in these vascular structures [36]. The localization of both ICAM‐2 and VE‐cadherin at HUVEC membranes indicates the establishment of intercellular junctions and a functional endothelial phenotype, supporting cell–cell adhesion, barrier integrity, and vascular organization in vitro. HUVECs were embedded within the layers of vimentin‐positive MSCs, suggesting close cellular interactions between endothelial and mesenchymal cells that may facilitate endothelial integration and support the formation of vascular‐like networks within the spheroids (Figure 4F and Figures S4C, S6B).

### 3.5 Endothelial Cells from Liver Spheroids form Anastomoses with External Endothelial Cells

**FIGURE 5:**
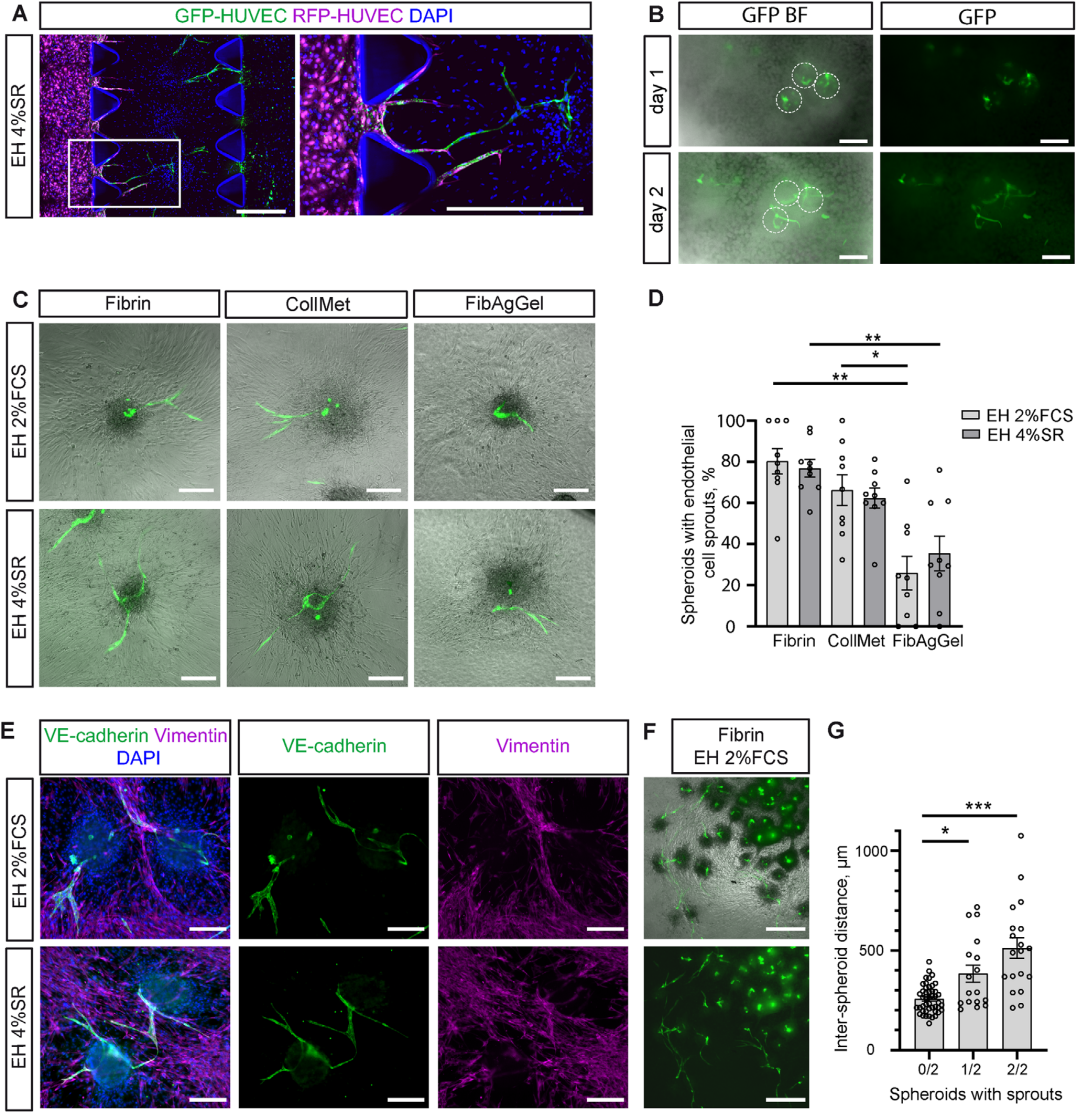
Triple‐cell liver spheroid‐derived HUVECs can form anastomoses with external endothelial channels in microfluidic devices, as well as with HUVECs from adjacent spheroids, and can extend into a host tissue. (A) RFP‐expressing HUVECs were loaded into the side channels of a microfluidic chip, and triple‐cell spheroids containing GFP‐HUVECs were embedded into fibrin in the main chip body. Native RFP signals, signals obtained with anti‐GFP antibody and DAPI are shown in magenta, green, and blue, respectively. The right image represents a magnified area of the labeled area of the left image. Scale bar: 500 µm. (B) GFP fluorescence microscopy images (right), merged with bright‐field images (left) of mouse liver explants grafted with 4‐day‐old triple‐cell spheroids on days 1 (top) and 2 (bottom) after injection. The grafted spheroids are marked with dashed circles. Scale bar: 200 µm. (C) GFP fluorescence microscopy images merged with bright‐field images of triple‐cell spheroids cultured in EH 2%FCS or EH 4%SR, embedded in indicated hydrogels on day 2 post‐embedding. Scale bar: 200 µm. (D) Percentage of triple‐cell spheroids cultured in EH 2%FCS or EH 4%SR that have formed endothelial cell sprouts on day 3 after embedding in the indicated hydrogels, three biological replicates with three technical replicates each (*n* (fibrin, EH 2%FCS) = 232 spheroids evaluated, *n* (fibrin, EH 4%SR) = 316, *n* (FibAgGel, EH 2%FCS) = 221, *n* (FibAgGel, EH 4%SR) = 206, (CollMet, EH 2%FCS) = 197, *n* (CollMet, EH 4%SR) = 132). Each dot represents one experiment. The Games–Howell's multiple comparisons test, which accounts for unequal variances and sample sizes, was applied to determine whether the datasets were significantly different. (E) Representative immunofluorescence images of triple‐cell spheroids embedded in fibrin gel, cultured in EH 2%FCS or EH 4%SR, on day 2 after embedding, and stained for VE‐cadherin (green) and vimentin (magenta). DAPI staining is shown in blue. Scale bar: 200 µm. (F) Images of GFP fluorescence (bottom) and merged with bright field (top) of triple‐cell spheroids cultured in EH 2%FCS on day 3 post‐embedding into fibrin gel. Scale bar: 500 µm. (G) Number of spheroids with endothelial cell sprouts and respective inter‐spheroid distance (defined as the distance between the centers of two spheroids). The triple‐cell spheroids were cultured in EH 2%FCS and analyzed on day 3 post‐embedding into fibrin gel. For each spheroid pair, it was noted whether neither spheroid (0/2), only one spheroid (1/2) or both spheroids (2/2) exhibited sprouts. *n* = 82 spheroid pairs. To determine whether the datasets were significantly different, the Kruskal–Wallis test was applied. (D, G): Means ± SEM are shown; individual data points are indicated. *p ≤ 0.05, **p ≤ 0.01, ***p ≤ 0.001.

To use triple‐cell spheroids as building blocks for the generation of larger tissue formations, continuous perfusion through the entire tissue must be achieved. As proof‐of‐compatibility with perfusable systems, we assessed if the integrated endothelial cells within spheroids can in principle form anastomoses with external endothelial networks by positioning vascularized spheroids next to endothelialized channels in microfluidic chips. GFP‐positive HUVECs from spheroids established connections with vascular sprouts formed by RFP‐positive HUVECs extending from the surrounding endothelial channel and migrated toward the root of the chip‐grown sprout, indicating the formation of a stable connection (Figure 5A and Figure S7); 35.7% (5/14) and 26.9% (7/26) of the spheroids formed anastomoses with the parent vessel in case of spheroids cultured in EH 2%FCS and EH 4%SR, respectively, whereas 14.3% (2/14) and 42.3% (11/26) of the spheroids formed sprouts directed toward the parent channels but did not reach the parent vessel within the 5‐day observation period when generated and cultured in EH 2%FCS and EH 4%SR, respectively. Vessels formed by anastomosis of spheroid‐derived HUVECs with the endothelial cells from the parent channel could be perfused with 1 µm fluorescent beads (FluoSpheres), showing perfusability and flow extending into the GFP‐positive regions of the vessel (Figure S7). This indicates that the endothelial cells within the spheroid can integrate into external vascular networks, thereby demonstrating their potential to contribute to continuous microvascular structures.

### 3.6 Vascular Structures Formed Within the Triple‐Cell Liver Spheroids Extend into Mouse Liver Tissue Upon Grafting

To explore the compatibility of the triple‐cell spheroids with a native tissue environment, we next aimed to evaluate whether vascular structures formed within the triple‐cell liver spheroids could invade a host tissue following grafting. To this end, triple‐cell spheroids composed of hepatocytes, GFP‐labeled HUVECs, and mesenchymal stem cells were implanted into mouse liver explants from postnatal day 0 (P0) mice. The spheroid‐derived GFP‐HUVECs continued to self‐organize into vascular‐like structures extending into the host parenchyma (Figure 5B).

### 3.7 Liver Spheroids as Building Blocks for the Formation of Larger Vascularized Tissue

We next asked if endothelial cell connections could be formed between multiple adjacent vascularized liver spheroids. This would serve as proof of principle that these spheroids enable a rapid intra‐tissue vascularization and would underscore their principal applicability as building blocks to assemble larger tissue formations. For this purpose, endothelial cell sprouting between the spheroids had to be facilitated. Initial experiments with spheroids randomly placed in different types of hydrogels yielded several important results. First, among the three hydrogels tested (fibrin, collagen‐methylcellulose (CollMet), fibrin‐agarose‐gelatin blend (FibAgGel)), fibrin and CollMet gels proved most effective, yielding the highest number of spheroids with sprouts (Figure 5C,D). There was a trend toward higher maximal sprout length in fibrin and CollMet gels compared with FibAgGel, reaching statistically significant differences in case of fibrin vs. FibAgGel in medium EH 2%FCS and CollMet vs. FibAgGel in EH 4%SR (Figure S8). Mechanical characterization of the hydrogels revealed that all three are very soft and fall within the range of human liver tissue (Figure S9) [37]. The lower number of spheroids with endothelial sprouts in FibAgGel is consistent with endothelial cells not binding and not degrading agarose [38]. Both processes are required for endothelial cell sprouting.

The second important observation was that the migration of spindle‐shaped GFP‐negative cells out of the spheroid often preceded the appearance of GFP‐positive endothelial cell sprouts. Immunostaining of the fibrin gel‐embedded spheroids revealed vimentin‐positive MSCs in the areas surrounding the spheroids, while VE‐cadherin‐positive endothelial cells extended toward these MSCs (Figure 5E). Furthermore, endothelial sprouts occurred mainly at spheroid sites where the distance to neighboring spheroids was larger: no sprouts were detected between spheroids placed less than 200 µm apart, and the permissive distance lied between 205 and 1075 µm (Figure 5F,G).

**FIGURE 6:**
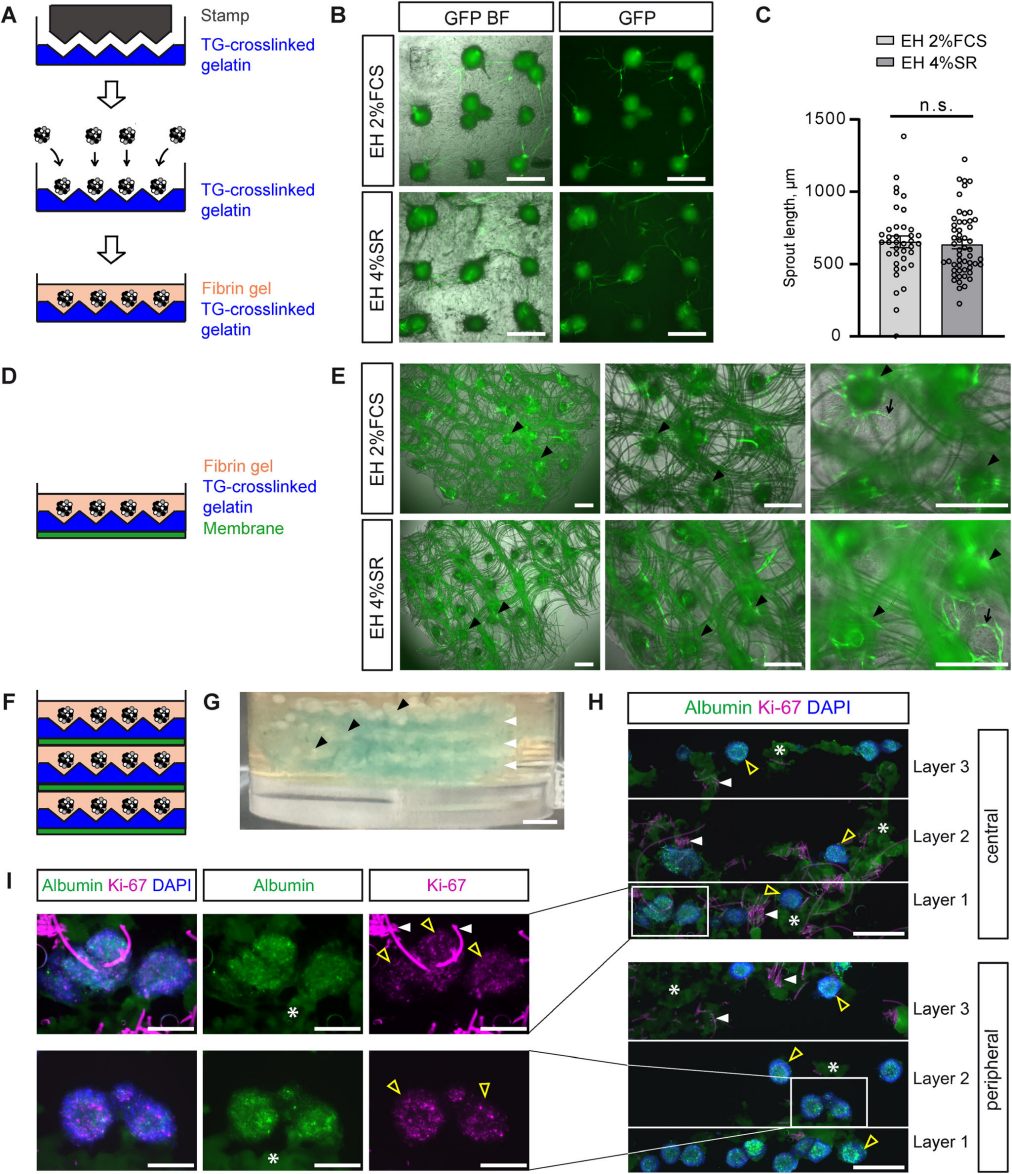
Use of the triple‐cell spheroids as building blocks to form single layer and multilayer tissue constructs. (A) Schematic workflow of scalable spheroid embedding with defined inter‐spheroid space. A thin gelatin layer with microwells is cast using a negative silicone stamp. After gelation, the gelatin is cross‐linked with transglutaminase. Next, spheroids are loaded into the microwells, and fibrin gel is added while ensuring a proper spheroid positioning in the microwells. (B) GFP fluorescence microscopy images (right) and merged with bright field (left) of evenly spaced triple‐cell spheroids, cultured in EH 2%FCS or EH 4%SR on day 13 post‐embedding into fibrin gel. Scale bar: 500 µm. (C) Lengths of the longest sprouts of endothelial cells from positioned triple‐cell spheroids, cultured in EH 2%FCS or EH 4%SR on day 13 post‐embedding into fibrin gel. The sprout lengths, defined as the linear distances from the spheroid center to the end of a sprout, were measured for all positioned triple‐cell spheroids in a 3 × 3 spheroid matrix (as shown in (B)). Three biological replicates; *n* (EH 2%FCS) = 38, *n* (EH 4%SR) = 53. An unpaired *t*‐test with Welch's correction was applied. Means ± SEM are shown; individual data points are indicated. n.s. ‐ non‐significant. (D) Schematic representation of the single layer spheroid embedding with defined inter‐spheroid space on a PGA membrane. (E) Fluorescent microscopy images merged with bright field showing triple‐cell spheroids cultured in EH 2%FCS or EH 4%SR on day 6 post‐embedding into a single layer of fibrin gel cast over gelatin microwells on top of a PGA membrane (as shown in D). Note the autofluorescence of membrane fibers. Black arrowheads point to corresponding single spheroids in all three magnifications. Black arrows mark endothelial sprouts. Scale bar: 500 µm. (F) Schematic representation of a stacked triple layer tissue construct. (G) Photograph of a triple layer tissue construct consisting of evenly distributed gel‐embedded spheroids in a cell culture plate with white arrowheads indicating the incorporated PGA membrane (turquoise). Black arrowheads point to single spheroids in each layer. Scale bar: 1 mm. (H) Representative immunofluorescence images of central and peripheral cryosections (approximately 1.8 and 0.8 mm from the outer edge, respectively) of a triple layer tissue construct 2 days after stacking, stained for albumin (green) and Ki‐67 (magenta). DAPI staining is shown in blue. Yellow hollow arrowheads indicate spheroids in each layer. White asterisks indicate green immunosignals in the gel. White arrowheads mark autofluorescent membrane fibers (magenta). Scale bar: 500 µm. (I) Higher magnification of the boxed area of the immunofluorescence microscopy images in (H), showing albumin (green) and Ki‐67 (magenta) immunosignals of spheroids. DAPI staining is shown in blue. White asterisks indicate green immunosignals in the gel. Yellow hollow arrow heads indicate spheroids. White arrowheads mark autofluorescent membrane fibers (magenta). Scale bar: 200 µm.

In final experiments, the inter‐spheroid sprouting of endothelial cells was studied and exploited to generate a tissue layer composed of multiple vascularly interconnected spheroids. Based on the previously derived results (Figure 5C,D,F,G) that provided suitable distances between spheroids and permissive hydrogels, we considered the following to fabricate one tissue layer assembled from multiple vascularized liver spheroids interconnected by endothelial cells: spheroids should be evenly spaced, with a distance of 200–1000 µm between neighboring spheroids, and bridges of either fibrin or CollMet gel should facilitate endothelial cell sprouting between them. To ensure even spacing between spheroids, we fabricated a negative silicone stamp to cast a thin layer of gelatin with evenly distributed wells for spheroid placement (Figure 6A), leading to an inter‐spheroid distance of 839 ± 34 µm horizontally and vertically, and 982 ± 20 µm diagonally (both means ± SEM). Fibrin gel containing spheroids was pipetted onto the gelatin wells, ensuring an even distribution of 1–3 spheroids per well and the formation of fibrin bridges between the spheroid‐containing wells (Figure 6A). In this setup, the gelatin wells served two functions: (1) They could be readily cast with defined size and distance, which was essential for the controlled seeding of spheroids at the beginning of the experiment. (2) During cultivation of the spheroids, the gelatin base mechanically reinforced the surrounding fibrin matrix. To maintain structural support, the gelatin matrix was cross‐linked using transglutaminase. This approach ensured that spheroids remained confined within their designated wells and maintained defined inter‐spheroid spacing throughout the culture period. Without transglutaminase treatment, the patterned gelatin transitioned from the gel to the sol state and dissolved under cell culture conditions. As a result, shrinkage of the remaining fibrin gel could be observed, which – without the previous mechanical support through the gelatin matrix – could not withstand the contractile forces of the embedded cells, a frequently reported challenge using this protein‐based hydrogel in high‐density 3D cell cultures. The shrinkage occurring in the absence of transglutaminase treatment altered the well geometry, led to irregular spheroid positioning, and caused a loss of defined spheroid spacing (Figure S10), thereby compromising the reproducibility of the experimental setup. The described methodology was then applied to study the formation of endothelial sprouts between spheroids under controlled spatiotemporal conditions. The first sprouts appeared 5–7 days after embedding, and vascular inter‐connections had formed 9–13 days post‐embedding (Figure 6B,C), as evidenced by the fusion of GFP‐HUVEC sprouts from neighboring spheroids. The endothelial sprouts had a length of 654 ± 41 and 636 ± 31 µm (both means ± SEM) in EH 2%FCS and EH 4%SR, respectively, with no significant differences observed between the two studied media compositions (Figure 6C).

To provide further stability and allow easy manipulation of tissue layers, we introduced a porous biocompatible membrane made of polyglycolic acid (PGA) as a base (Figure S11) on which gelatin wells were cast (Figure 6D). The membrane's high porosity promotes the exchange of substances between the cell culture medium and the spheroids, and its biodegradability should in principle enable a transition into an entirely cellular construct in the long term. The planar layers of spheroids embedded on top of this membrane were cultured for one week to form sprouts between neighboring spheroids, after which single layers were stacked to achieve a larger tissue construct with prospective physiological thickness (Figure 6E,F,G). Spheroids in all three layers, and in both peripheral and central regions of the tissue construct contained albumin‐producing and proliferating cells (Figure 6H,I). These results prove the general feasibility of using vascularized liver spheroids as building blocks to generate vascularized single layer or multilayer tissue constructs.

## 4 Discussion

Vascularized human liver spheroids hold great potential in the hepatology field: as tissue models to study liver diseases, assess substance toxicology, and develop drugs, as well as tissue building blocks to rapidly fabricate larger tissue units that can replace or support lost or dysfunctional human liver tissue. Our study identifies key parameters that influence the coordination of multiple self‐organizing processes that occur simultaneously in space and time upon the aggregation of different cell types into spheroids, ultimately resulting in the formation of vascularized human liver tissue. We furthermore present a new methodology that enables the formation of larger, contiguous, vascularized tissue formations from spheroids by creating conditions for inter‐spheroid vascular connections and layer‐by‐layer fabrication.

Our work outlines the key requirements for establishing long‐term cultures of self‐assembled vascularized liver spheroids. (i) The inclusion of a mesenchymal cell type into spheroids at specific cell ratios prevents individual liver and endothelial cells from forming two spatially separated, aggregated cell groups, with central and peripheral spheroid localization, respectively, and enables the endothelial cells to organize themselves into network‐like structures. Mesenchymal cells, including adipose tissue‐derived mesenchymal stem cells as used in our study, are known to support tissue vascularization [39, 40, 41, 42]. MSCs are recruited onto endothelial tubes and form a pericyte‐like coverage [43] that could provide pericyte‐like physical support and improve vessel stability. They promote the formation of endothelial cell self‐organization into vascular structures by secreting pro‐angiogenic factors, and by producing and remodeling extracellular matrix. In the vascularized liver spheroids presented here, endothelial cells were found to be embedded within the layers of vimentin‐positive MSCs and in collagen‐rich areas, consistent with this endothelial cell‐supporting function of MSCs. (ii) One numerical ratio of these three cell types (5:2:2 of HepaRG:GFP‐HUVEC:MSC) leads to consistent vascularized spheroid formation and at the same time a high proportion of hepatocytes. (iii) A critical challenge of maintaining spheroids consisting of cells that are typically grown in very different media formulations is to identify a common medium that accommodates the requirements of all included cell types. For liver spheroids based on HepaRG cells and HUVECs, we show that under the described serum‐reduced and serum‐free culture conditions, the HepaRG‐derived hepatocytes exhibit a metabolically active, relatively mature phenotype according to hepatocyte marker expression, functional assays, and transmission electron microscopy. (iv) Moreover, these culture conditions support the development of endothelial cell networks with the potential of lumen formation. Importantly, we identified endothelial cell formations with ultrastructural features of capillaries. This result is a notable achievement since for the majority of reported liver spheroids, lumina only form upon transplantation [16, 17, 18], and of the ones describing lumina, we are only aware of one group that has provided ultrastructural images of formed vessels [20, 21]. In contrast to our work, the cells used in these two studies were derived from human embryonal and human induced pluripotent stem cells.

Although direct comparison of vascular and hepatic parameters between the spheroids of this study and previously published liver organoids with a lumen‐containing vascular system is challenging due to differing analysis methods, we would like to highlight several aspects. The fraction of hepatic cells in spheroids cultured in EH 2%FCS and EH 4%SR (mean values of E‐cadherin‐positive cells: 57%–68%) is higher than the one quantified by Kim et al. (less than 50%) [44]. On the functional level, we show that the integration of vasculature into HepaRG spheroids overall does not interfere with urea production, CYP3A4 activity, and albumin secretion in vascularized liver spheroids in comparison to spheroids consisting of HepaRG cells only. The triple‐cell spheroids exhibit relatively high albumin production and comparatively low urea secretion, consistent with published data on HepaRG cells [45, 46]. In terms of vascular structures, the median lumen diameter of 8.3 µm reported by Harrison, Siller, Tanaka, Chollet et al. [19] and the values presented in our study (mean lumen diameter of 5.34 and 5.65 µm, maximum lumen diameter of 11 and 11.1 µm) are within the range of capillaries.

Based on our results, we suggest that the described approach may serve as a template for generating other human spheroids consisting of an organ‐specific cell type, endothelial cells, and a mesenchymal cell type, namely to apply a 1:1 mixture of basal media used for endothelial cells and the organ‐specific cell type supplemented with 100% final concentrations of all additives required for the two cell types, with a potential use of KnockOut Serum Replacement instead of FCS. Importantly, our spheroid generation and maintenance protocol does not necessitate any additional extracellular matrix or hydrogel and is easily scalable.

Most studies on spheroids as tissue building blocks have focused on fabrication methods that promote a narrow spheroid arrangement, facilitating fusion between neighboring spheroids. However, in case of vascularized spheroids, such short inter‐spheroid distance might be disadvantageous. We demonstrate that the distance between spheroids is a critical factor for the formation of endothelial connections between vascularized liver spheroids, and that spheroids placed in close proximity fail to establish vascular interconnections. These results are consistent with the findings of Kim et al. [44] who demonstrated that a distance of 200 µm between spheroids generated from MSCs derived from human adipose tissue, alone or in combination with HUVECs, was required for MSC migration into the surrounding Matrigel and for the formation of cellular bridges to neighboring spheroids [44]. Similar to Kim et al. [44], we show that MSCs appear to act as guiding cues for endothelial outgrowth, supporting their directed outgrowth toward adjacent spheroids. These observations challenge the prevailing aim to achieve larger tissue formations by tightly packing spheroids as tissue building blocks. Instead, they underscore the need for automated technologies that allow precise positioning of spheroids with defined inter‐spheroid distance. This approach could provide an additional advantage, namely the possibility of functionalizing the inter‐spheroid space, for example by incorporating biomaterials or supportive cell types to further promote vascular integration and tissue function. In addition, spheroid placement strategies, including the microneedles‐based (“Kenzan”) spheroid assembly method [47], could benefit from the acquired insights on spheroid spacing to facilitate inter‐spheroid vascular connections. Irrespective of the fabrication method, creating spaces between adjacent spheroids reduces the required spheroid number, and using prefabricated spheroid‐containing gel layers can accelerate the fabrication process. Ultimately, leaving space between spheroids as modular building blocks should allow the construction of tissues with cell densities approaching those of native organs, since spheroids inherently contain a high cellular density.

We provide a methodology that allows the production of a tissue layer of vascularly connected liver spheroids, which involves simply seeding prefabricated spheroids within a fibrin gel into a hydrogel layer with cavities, that are quickly generated using a stamp. In addition, we demonstrate that using a biodegradable membrane made of PGA as a dimensionally stable bottom structure in combination with the resulting plane fibrin surface makes this method amenable to simple and rapid layer‐by‐layer fabrication. An advantage of the presented process is that individual vascularized layers can be created, maintained, and monitored separately and only stacked after endothelial connections between the spheroids have formed. Combining self‐organization of multiple cell types into vascularized liver spheroids with the presented methodology may enhance the in vitro survival of engineered tissue constructs and may open new ways to their fabrication.

Future investigations should explore the inclusion and maintenance of additional cell types, such as immune cells, either within spheroids or in the inter‐spheroidal space, according to the protocols and cell culture conditions described here. Moreover, the in vitro perfusability of tissue constructs engineered in this way should be assessed.

For in vivo applications, that depend on rapid vascularization upon implantation, the demonstrated capacity of the human spheroid‐derived endothelial structures to extend into a physiological microenvironment, and our approach to generating vascularly interconnected tissue blocks, may improve the survival of transplanted tissue built from spheroids or organoids.

## Author Contributions

U.A.N., A.B., H.G., and A.F. contributed to conceptualization. A.F., O.V.D., T.I.M., and J.G. conducted the formal analysis. U.A.N., A.B., and H.G. contributed to funding acquisition. A.F., O.V.D., B.K.S., J.B.S., and T.I.M. performed the investigation. U.A.N., A.F., O.V.D., J.B.S., T.I.M., B.K.S., K.M., J.G., L.K., H.G., and A.B. contributed to methodology. U.A.N. carried out project administration. U.A.N., H.G., B.K.S., and A.B. contributed to resources. U.A.N. conducted supervision. A.F., O.V.D., B.K.S., J.B.S., and J.G. performed visualization. U.A.N. and A.F. contributed to writing the original draft. A.F., O.V.D., J.B.S., T.I.M., J.G., A.B., B.K.S., H.G., and U.A.N. contributed to the reviewing and editing of the manuscript.

## Funding

We kindly acknowledge the financial support by the Merck Sustainability Hub, the LOEWE Research Initiatives Network by the German federal state of Hesse (research cluster FlowForLife, reference number LOEWE/2/14/519/03/07.011(0002)/78), and the Dr. Illing foundation.

## Ethics Statement

All animal procedures were performed in compliance with German national guidelines and approved by the local authorities (approval for the conduct of scientific experiments DA8/2000, Regierungspräsidium Darmstadt, Hesse, Germany).

## Conflicts of Interest

A patent has been filed (U.A.N., O.V.D.). B.K.S. received funds from Bayer Healthcare, Astra Zeneca, medupdate, Boehringer Ingelheim and Bristol Myers Squibb as well as from the IMPP. In addition, B.K.S. holds a research cooperation with Resolve Biosciences.

## Supporting information




**Supporting File: 1** smll74536‐sup‐0001‐SuppMat.pdf

**Supporting File: 2** smll74536‐sup‐0002‐VideoS1.avi

**Supporting File: 3** smll74536‐sup‐0003‐VideoS2.avi

## Data Availability

The data that support the findings of this study are available from the corresponding author upon request.
