# Supplementary material for: Self‐Organized Vascularized Human Liver Spheroids: Serum‐Free Culture Conditions and Use as Tissue Building Blocks
Source: Small. 2026 Jul 19;22(49):e74536. doi: 10.1002/smll.74536 (PMC13533262; doi:10.1002/smll.74536)
Supplement: Supplementary file 1 [file SMLL-22-e74536-s001.pdf]

Supplemental data

Self-organized vascularized human liver spheroids: Serum-free culture conditions and use as tissue building blocks

Alina Filatova[1], Olivera Valentirović Dann[1], Jennifer B. Schwarzkopf[2,7,8], Tengku I. Maulana[2,7], Jamina S. Gerhardus[3], Leon Kaysan[1], Katja Meier[2], Andreas Blaeser[3,4], Beate K. Straub[5,6], Holger Gerhardt[2,7,8,9], Ulrike A. Nuber[1,4,10*]

[*]corresponding author

[1] Stem Cell and Development Biology, Department of Biology, Technical University of Darmstadt, Germany

[2] Max Delbrück Center for Molecular Medicine in the Helmholtz Association, Berlin, Germany

[3] Institute for BioMedical Printing Technology, Department of Mechanical Engineering, Technical University of Darmstadt, Germany

[4] Centre for Synthetic Biology, Technical University of Darmstadt, Germany

[5] Tissue Biobank of the University Medical Center Mainz, Germany

[6] Institute of Pathology, University Medical Center of the Johannes Gutenberg-University Mainz, Germany

[7] DZHK (German Center for Cardiovascular Research), Berlin, Germany

[8] Charité - Universitätsmedizin Berlin, Berlin, Germany

[9] Berlin Institute of Health, Berlin, Germany

[10] Department of Mechanical Engineering, Technical University of Darmstadt, Germany

Supplemental Videos S1, S2. Representative confocal z-stacks of triple-cell spheroids cultured for 14 days in EH 2% FCS (S1) or EH 4% SR (S2) medium, showing the three-dimensional organization of GFP-HUVEC cells and MSC cells. Whole-mount immunofluorescence staining of the spheroids was performed using antibodies against VE-cadherin (shown in green; labels GFP-HUVEC) and vimentin (shown in white; labels GFP-HUVEC and MSC). Note the lumen formed by GFP-HUVEC cells.

Table S1. Primary antibodies and conditions used in immunostainings.

**Primary antibody**	**Source**	**Dilution**
Albumin	A80-229F, Bethyl (Montgomery, Texas, USA)	1:200
Arginase	66129-1-Ig, Proteintech (Martinsried, Germany)	1:200
Cleaved Caspase-3 (Asp175)	9661, Cell Signaling Technology (Leiden, The Netherlands)	1:100
CYP3A4	MA5-17064, Thermo Fisher Scientific	1:100
E-cadherin	AF648, R&D Systems	1:400
GFP	ab13970, Abcam	1:300
HNF4α	3113, Cell Signaling Technology	1:200
ICAM-2	13355, Cell Signaling Technology	1:200
Ki67	ab16667, Abcam (Waltham, Massachusetts, USA)	1:100
Perilipin 2	610102, Progen Biotechnik (Heidelberg, Germany)	undiluted
Smooth muscle actin	IR61161-2, Agilent	undiluted
VE-cadherin	AF938, R&D Systems (Minneapolis, Minnesota, USA)	1:100
Vimentin	5741, Cell Signaling Technology	1:200

Table S2. Secondary antibodies and conditions used in immunostainings.

**Secondary antibody**	**Source**	**Dilution**
donkey anti-goat-AF488	705-545-147, Dianova	1:300
donkey anti-goat IgG	A11055, Invitrogen*	1:300
donkey anti-mouse-AF647	715-605-151, Dianova	1:300
donkey anti-mouse-AF647	A31571, Invitrogen*	1:300
donkey anti-mouse-Cy3	715-165-150, Dianova	1:300
donkey anti-rabbit-AF568	A10042, Invitrogen*	1:300
donkey anti-rabbit-AF647	711-605-152, Dianova	1:300
donkey anti-rabbit-Cy3	711-165-152, Dianova	1:300
donkey anti-chicken-AF488	703-545-155, Dianova	1:300

*Antibodies used in whole-mount immunofluorescent staining of spheroids for confocal microscopy

Table S3. Primers used in RT-qPCR.

**Target gene**	**Forward primer sequence (5‘ → 3‘)**	**Reverse primer sequence (5‘ → 3‘)**	**Amplicon size, bp**
CYP3A4	AAGAGCAACACAGAGCTGAAAG	AAGTCCATGTGAATGGGTTCCA	156
ALB	ATGCCTGCTGACTTGCCTTC	GCAGCAGCACGACAGAGTA	148
GLUL	TCTCCTCTCCTAACCTCGCTC	AAGTGGAACTTGCTGAGGT	99
HNF4A	ACATGTACTCCTGCAGATTTAG	CATAGCTTGACCTTCGAGTG	159
ASGR1	GGGAGGCAATGTGGGAAGAA	CAGGTCAGACACGAACTGCT	121
SLC10A1 (NTCP)	CCTCAAATCCAAACGGCCAC	GGCAGAGAGAACTGTGACGG	97
SLCO2B1 (OATP2B1)	TCCAGGAGCCCCTGAGAAGAT	TTCCTTGTCTGGTACCTGGGG	150
SLC2A2 (GLUT2)	GCCACACTCACACAAGACCT	AACTGGAAGGAACCCAGCAC	119
ABCC2 (MRP2)	CGTTCCAGACGCAGTCCA	TGCTCAAAACAAAGTGGCAGG	110
PECAM1	TGCAGTACACGGAAGTTCAAGT	GACAGCTTTCCGGACTTCAC	98
CDH5	CTCAGCCCACAGGCACG	CGTTGGGCAGGGTTAGCA	134
ICAM1	GGGAACAACCGGAAGGTGTA	GTTCCACCCGTTCTGGAGTC	136
YWHAZ	AGACGGAAGGTGCTGAGAAAA	ACCTCAGCCAAGTAACGGTAG	193
TBP	CTTGTGCTCACCCACCAAC	CTGCTCTGACTTTAGCACCTG	142

Supplemental figures

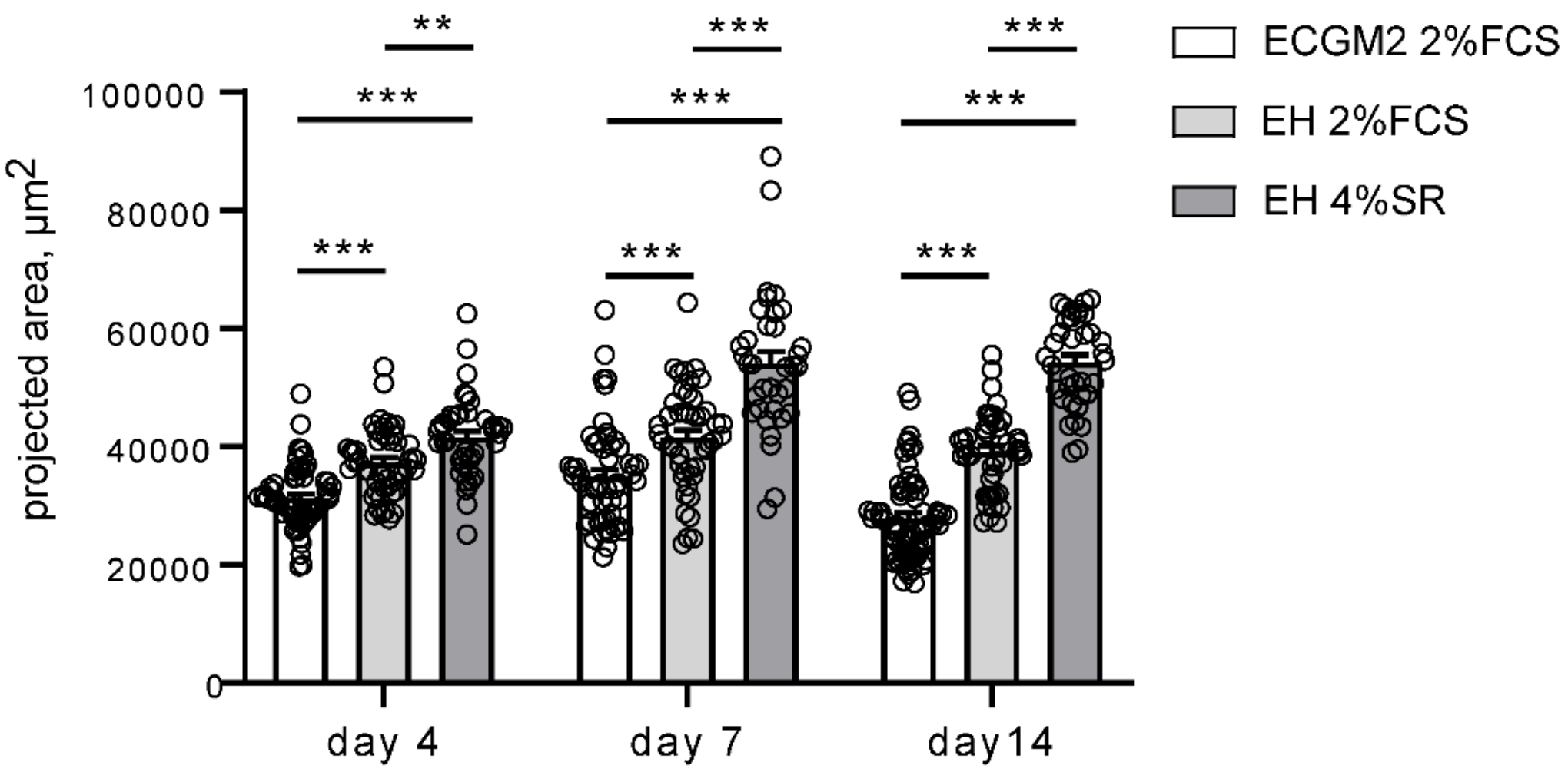

Figure S1. Graphical representation of Figure 2B, albeit with individual data points shown. Projected area of spheroids cultured in ECGM2 2%FCS, EH 2%FCS, or EH 4%SR on days 4, 7, and 14 after cell seeding. n = 35-69 spheroids per condition, three biological replicates. Means ± SEM are shown; individual data points are indicated. The Games-Howell's multiple comparisons test was applied to determine whether the following datasets were significantly different. ** $p \leq 0.01$, *** $p \leq 0.001$.

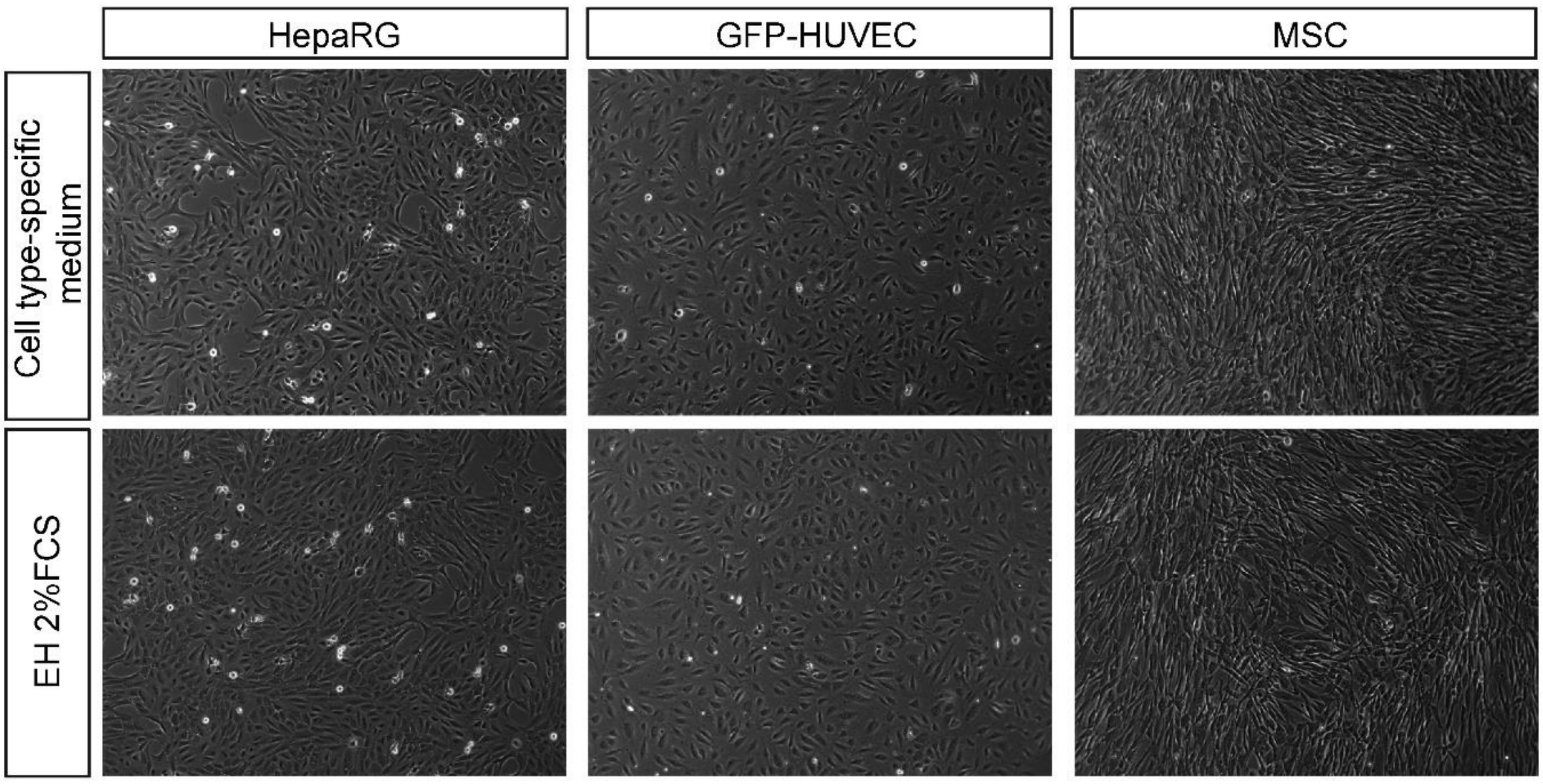

Figure S2. Bright-field microscopy images of HepaRG, GFP-HUVEC, and MSC cells cultured in their cell type-specific medium or in EH 2%FCS for 48h.

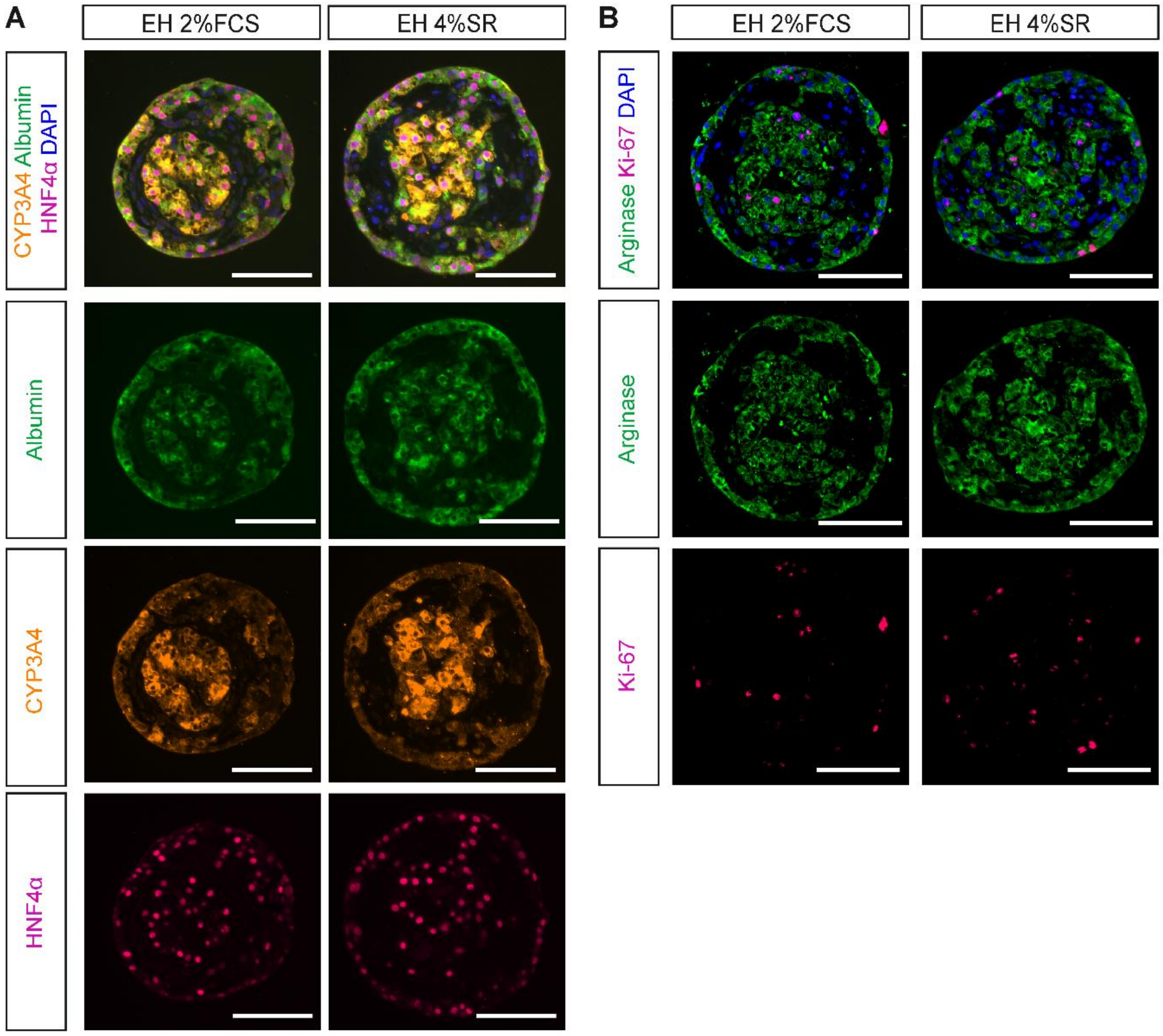

Figure S3. Hepatocyte markers in triple-cell liver spheroids cultured in EH 2%FCS or EH 4%SR for 14 days based on stainings of paraffin sections. A. Immunostainings with antibodies against albumin (green), CYP3A4 (orange), and HNF4α (magenta). DAPI staining is shown in blue. B. Immunosignals depicting the hepatocyte marker arginase (green) and the proliferation marker Ki-67 (magenta). DAPI staining is shown in blue. Scale bars: 100 µm.

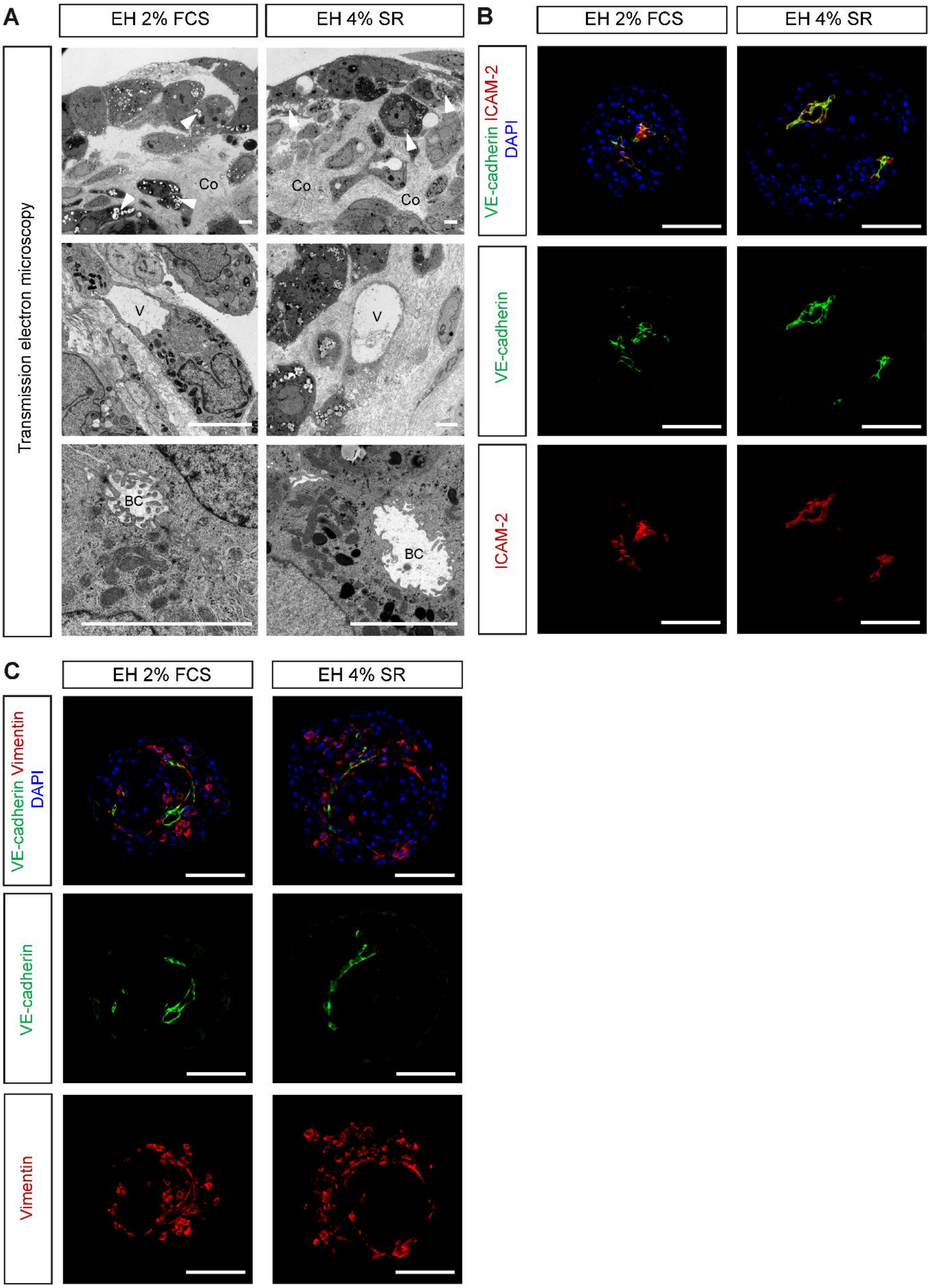

Figure S4. Transmission electron microscopy and immunofluorescence characterization of the endothelial structures within triple-cell liver spheroids. A. Transmission electron microscopy of tissue sections from spheroids cultured in EH 2%FCS or EH 4%SR for 14 days. Upper row:

hepatocytic cells at the outer region of spheroids with lipid droplets (white arrowheads) and collagenous stroma (indicated as “Co”). Middle row: capillary-type vessels (V). Bottom row: bile canaliculi (BC) formation in hepatocytic cells. Scale bars: 5 µm. (B,C). Vascular-like structures in spheroids cultured in EH 2%FCS or EH 4%SR for 14 days. Immunosignals of endothelial cell markers VE-cadherin (B, C, green) and ICAM-2 (B, red) and mesenchymal cell marker vimentin (C, red) in spheroid paraffin sections. DAPI staining is shown in blue. Scale bars: 100 µm.

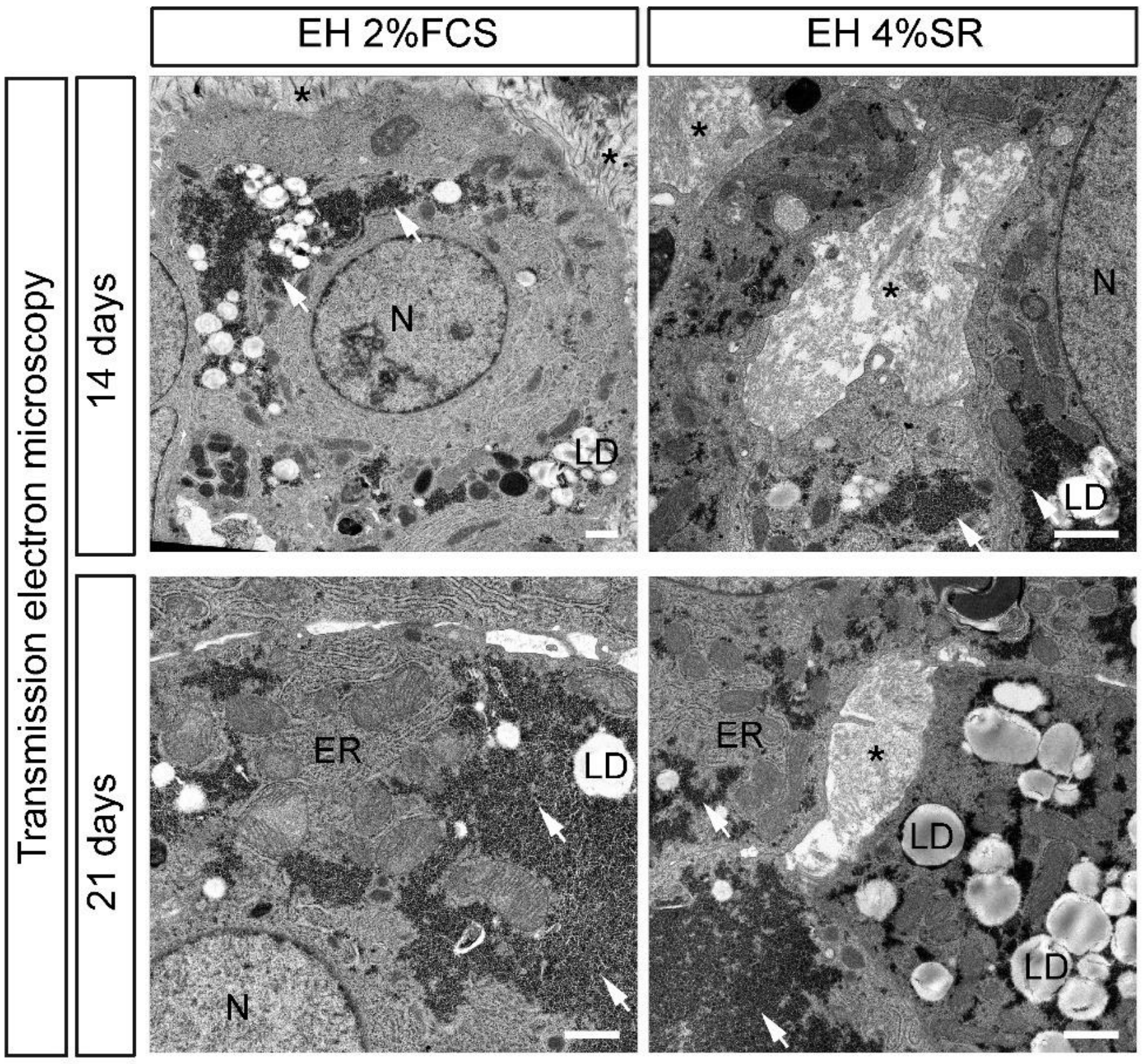

Figure S5. High-magnification transmission electron microscopy images of tissue sections from triple-cell liver spheroids cultured in EH 2%FCS or EH 4%SR for 14 or 21 days. The following structures are shown: cell nucleus (N), glycogen (white arrows), lipid droplets (LD), rough endoplasmic reticulum (ER), collagenous stroma (asterisk). Scale bars: 1 µm.

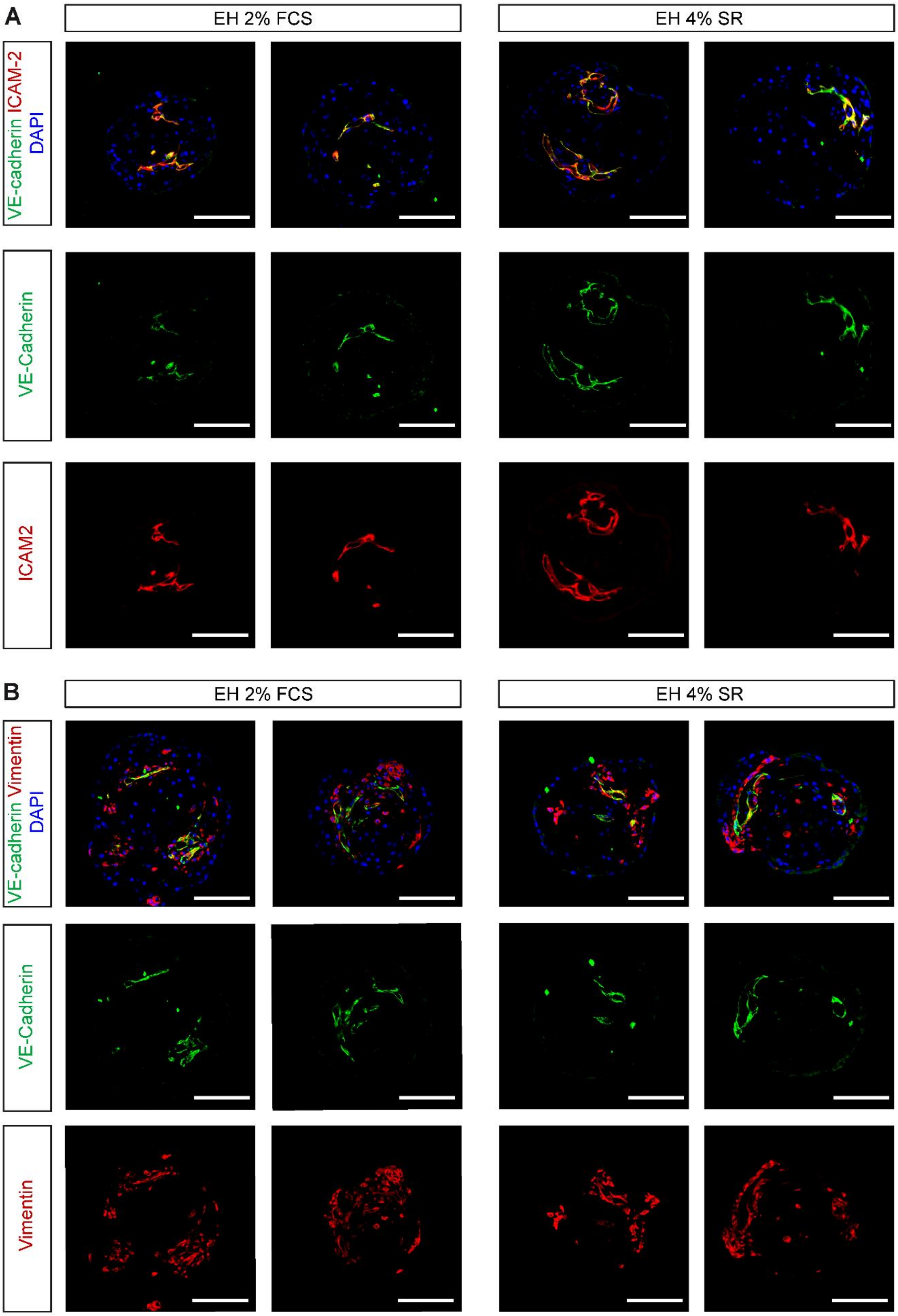

Figure S6. Additional examples of vascular-like structures in triple-cell liver spheroids cultured in EH 2%FCS or EH 4%SR for 21 days. Immunostainings of paraffin sections from spheroids

with antibodies against endothelial cell markers VE-cadherin (A, B, green) and ICAM-2 (A, red) and mesenchymal cell marker vimentin (B, red). Scale bars: 100 µm.

A B

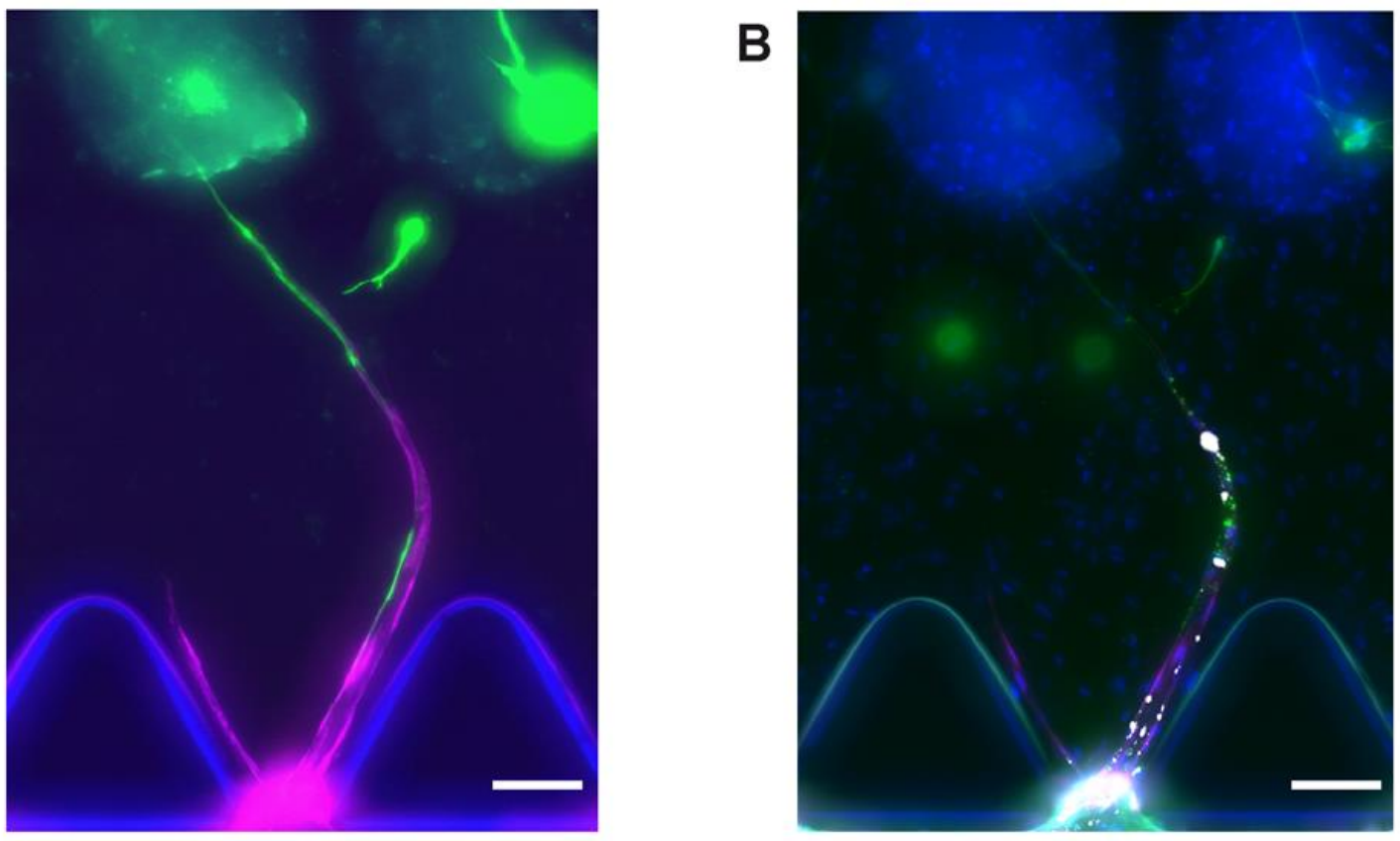

Figure S7. Perfusion of anastomoses between spheroid-derived vessels and vasculature-on-a-chip. A. Live imaging of the vascularized spheroid-on-a-chip system demonstrates anastomosis between the spheroid-derived GFP-HUVECs and RFP-HUVECs of the parent vessel. GFP-HUVECs migrate toward the root of the chip-grown sprout, indicating the formation of a stable connection. B. Fluorescent beads (1µm, FluoSpheres carboxylate-modified microspheres) were perfused through the parent vessel into the anastomosed vessel, showing perfusability and flow extending into the GFP-positive regions of the vessel. Scale bars: 100 µm.

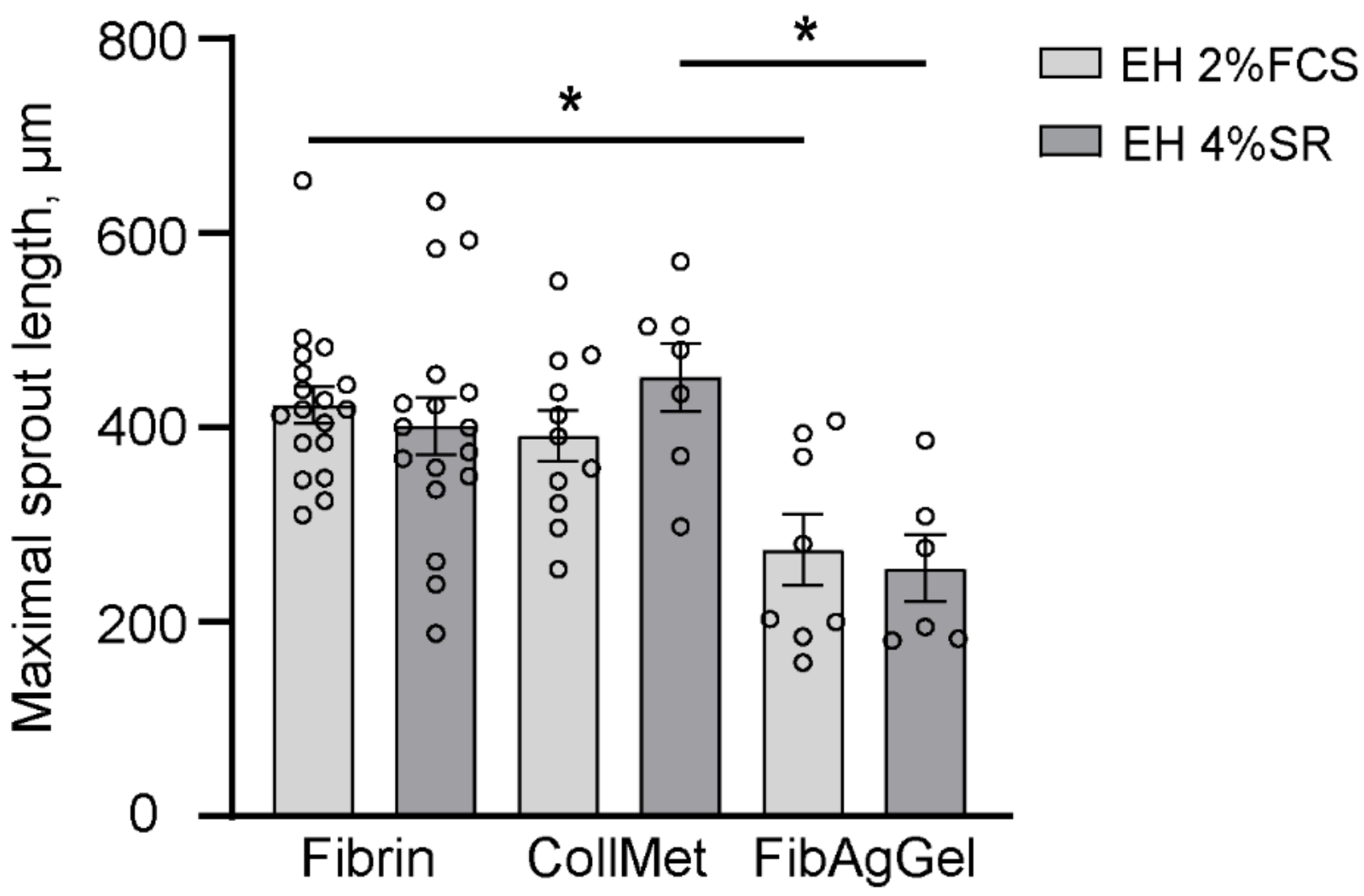

Figure S8. Maximal sprout length in triple-cell liver spheroids cultured in EH 2%FCS or EH 4%SR on day 3 after embedding in the indicated hydrogels. Sprout length was determined as the linear distance from the spheroid center to the end of a sprout. Means ± SEM are shown; individual data points are indicated. Experiments were done in three biological replicates with three technical replicates each, n (fibrin, EH 2%FCS) = 35, n (fibrin, EH 4%SR) = 22, n (FibAgGel, EH 2%FCS) = 8, n (FibAgGel, EH 4%SR) = 6, (CollMet, EH 2%FCS) = 17, n (CollMet, EH 4%SR) = 7. The Games-Howell's multiple comparisons test, which accounts for unequal variances and sample sizes, was applied to determine whether the datasets were significantly different. Significantly different values for each medium are marked; non-significant differences are not indicated. * $p \leq 0.05$.

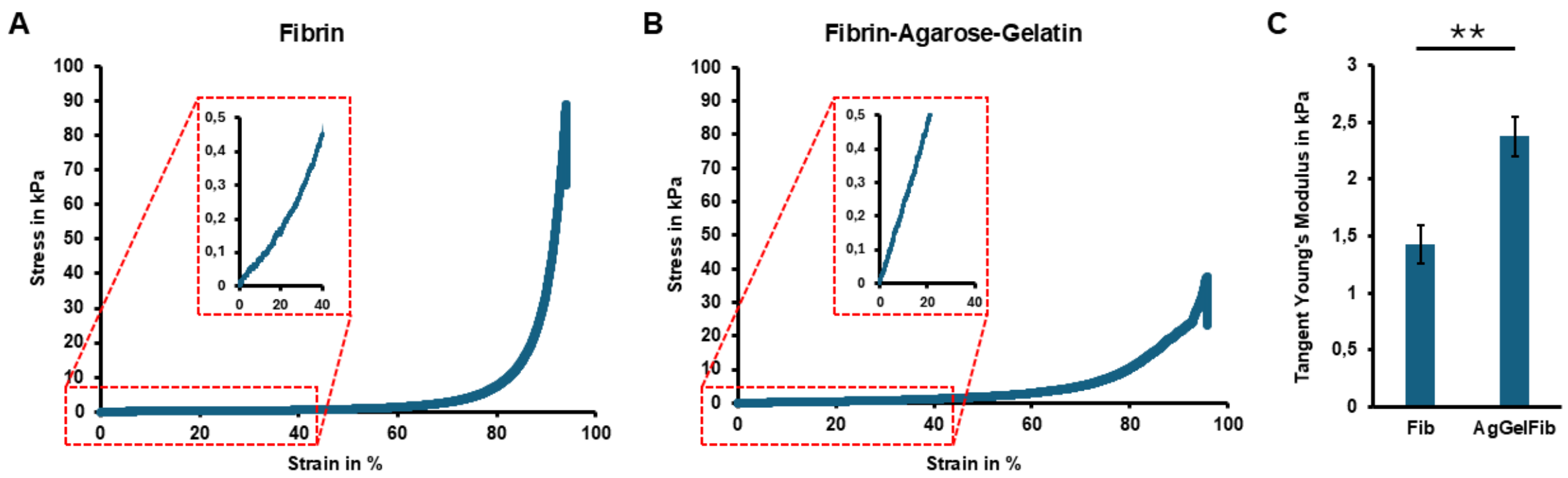

Figure S9. Mechanical characterization of fibrin and fibrin-agarose-gelatin blend. These tests could not be performed with collagen-methylcellulose (CollMet) samples due to their low level of crosslinking and the correlating low mechanical stiffness. (A) Exemplary stress-strain curve of fibrin with enlarged section of red framed area for better visualization of relevant data. (B) Exemplary stress-strain curve of fibrin-agarose-gelatin blend with enlarged section of red framed area for better visualization of the data. (C) Tangential Young's modulus calculated from compression tests for both hydrogels. Means ± SD are shown; n=3. ** $p \leq 0.01$.

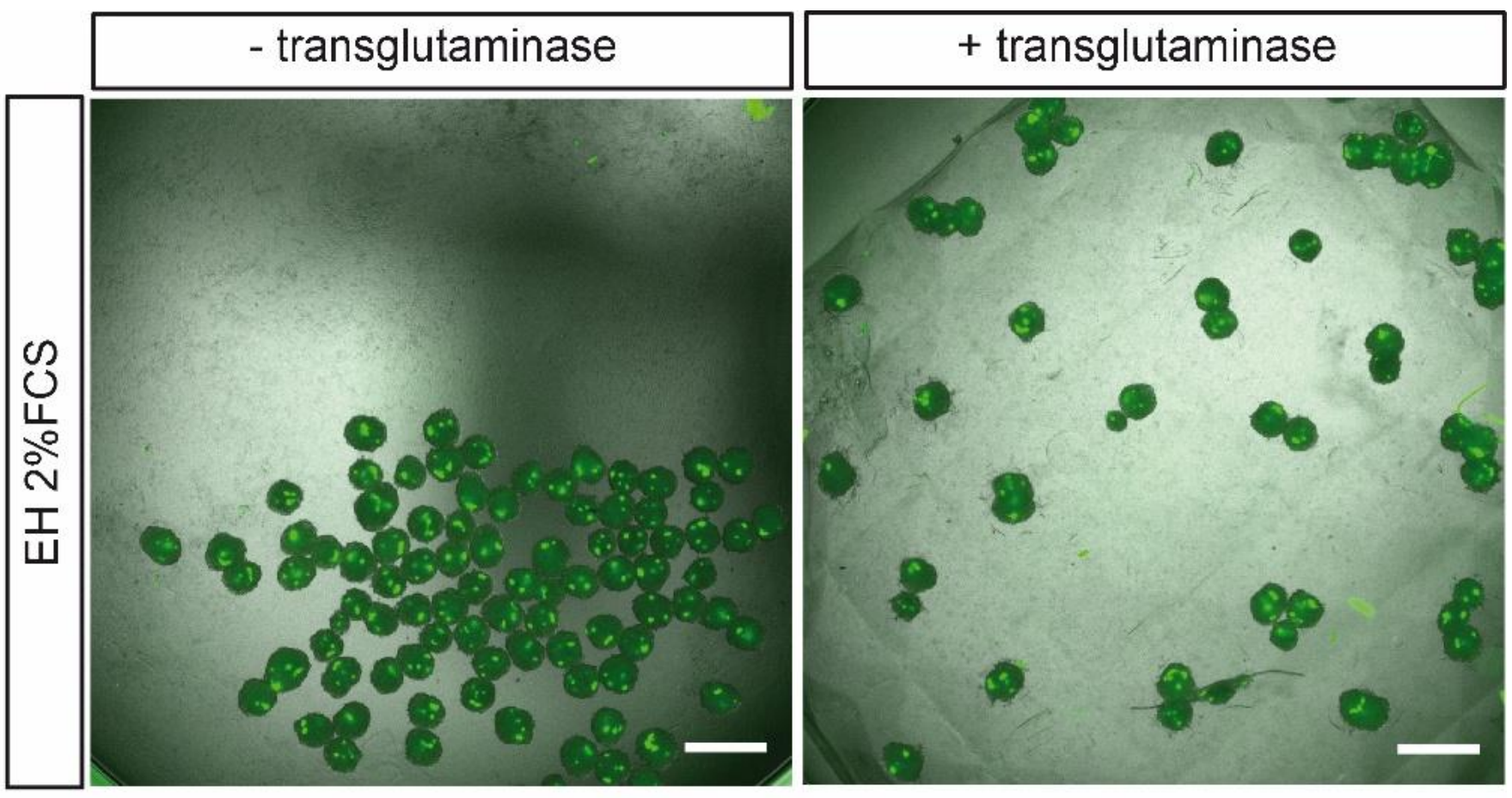

Figure S10. In the absence of trans-glutaminase treatment, gelatin shrinkage leads to an altered well geometry and a loss of defined spheroid spacing. Merged bright field and GFP fluorescence images of triple-cell liver spheroids cultured in EH 2%FCS right after embedding into fibrin gel and incubation at 37°C, without (left) and with (right) transglutaminase added to crosslink gelatin microwells. Scale bars: 500 µm.

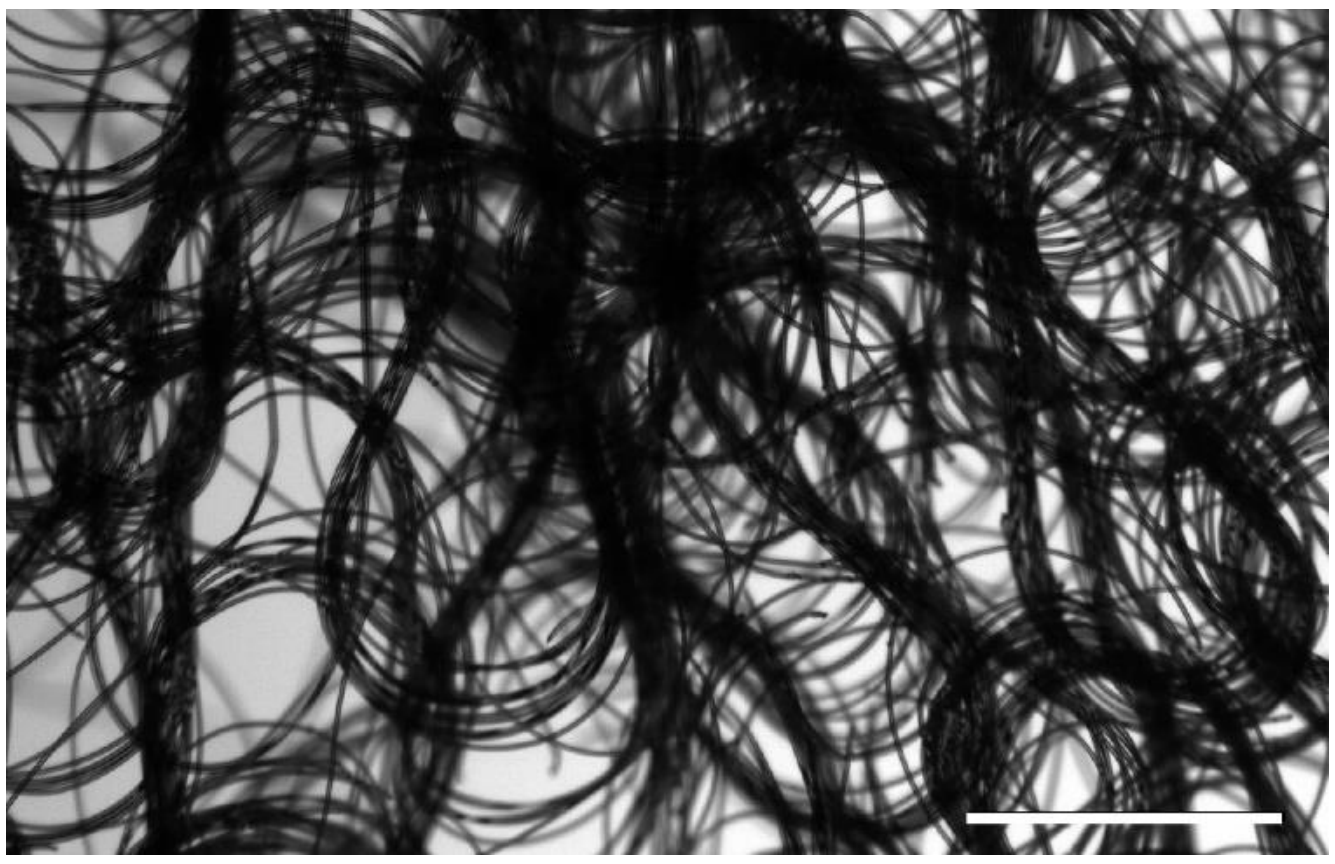

Figure S11. Bright-field image of the polyglycolic acid membrane Neoveil Mucosal Substitute sheet used to fabricate planar tissue layers. Scale bar: 1 mm.
